# Pearl millet [*Pennisetum glaucum* (L.) R. Br.] consensus linkage map constructed using four RIL mapping populations and newly developed EST-SSRs

**DOI:** 10.1186/1471-2164-14-159

**Published:** 2013-03-09

**Authors:** Vengaldas Rajaram, Thirunavukkarasu Nepolean, Senapathy Senthilvel, Rajeev K Varshney, Vincent Vadez, Rakesh K Srivastava, Trushar M Shah, Ambawat Supriya, Sushil Kumar, Basava Ramana Kumari, Amindala Bhanuprakash, Mangamoori Lakshmi Narasu, Oscar Riera-Lizarazu, Charles Thomas Hash

**Affiliations:** 1 International Crops Research Institute for the Semi-Arid Tropics (ICRISAT), Patancheru, Hyderabad, Andhra Pradesh 502 324, India; 2 Centre for Biotechnology, IST, JNTUH, Kukatpally, Hyderabad, Andhra Pradesh, 500 085, India; 3 Division of Genetics, Indian Agricultural Research Institute, New Delhi, 110 012, India; 4 Department of Crop Improvement, Directorate of Oilseeds Research, Hyderabad, 500 030, India; 5 Department of Biotechnology, College of Basic Sciences and Humanities, Chaudhary Charan Singh Haryana Agricultural University, Hisar, Haryana, 125 004, India; 6 Centre of Excellence in Biotechnology, Anand Agricultural University, Anand, Gujarat, 388 110, India; 7 Department of Crop and Soil Sciences, Oregon State University, Corvallis, Oregon, 97331, USA; 8 International Crops Research Institute for the Semi-Arid Tropics (ICRISAT), ICRISAT Sahelian Center, Niamey, BP 12404, Niger

**Keywords:** EST-SSR markers, EST, Linkage map, Consensus map, Drought stress, Pearl millet, Synteny

## Abstract

**Background:**

Pearl millet [*Pennisetum glaucum* (L.) R. Br.] is a widely cultivated drought- and high-temperature tolerant C4 cereal grown under dryland, rainfed and irrigated conditions in drought-prone regions of the tropics and sub-tropics of Africa, South Asia and the Americas. It is considered an orphan crop with relatively few genomic and genetic resources. This study was undertaken to increase the EST-based microsatellite marker and genetic resources for this crop to facilitate marker-assisted breeding.

**Results:**

Newly developed EST-SSR markers (99), along with previously mapped EST-SSR (17), genomic SSR (53) and STS (2) markers, were used to construct linkage maps of four F_7_ recombinant inbred populations (RIP) based on crosses ICMB 841-P3 × 863B-P2 (RIP A), H 77/833-2 × PRLT 2/89-33 (RIP B), 81B-P6 × ICMP 451-P8 (RIP C) and PT 732B-P2 × P1449-2-P1 (RIP D). Mapped loci numbers were greatest for RIP A (104), followed by RIP B (78), RIP C (64) and RIP D (59). Total map lengths (Haldane) were 615 cM, 690 cM, 428 cM and 276 cM, respectively. A total of 176 loci detected by 171 primer pairs were mapped among the four crosses. A consensus map of 174 loci (899 cM) detected by 169 primer pairs was constructed using MergeMap to integrate the individual linkage maps. Locus order in the consensus map was well conserved for nearly all linkage groups. Eighty-nine EST-SSR marker loci from this consensus map had significant BLAST hits (top hits with *e*-value ≤ 1E-10) on the genome sequences of rice, foxtail millet, sorghum, maize and *Brachypodium* with 35, 88, 58, 48 and 38 loci, respectively.

**Conclusion:**

The consensus map developed in the present study contains the largest set of mapped SSRs reported to date for pearl millet, and represents a major consolidation of existing pearl millet genetic mapping information. This study increased numbers of mapped pearl millet SSR markers by >50%, filling important gaps in previously published SSR-based linkage maps for this species and will greatly facilitate SSR-based QTL mapping and applied marker-assisted selection programs.

## Background

Pearl millet [*Pennisetum glaucum* (L.) R. Br.] is a highly cross-pollinated monocot belonging to the *Poaceae*. It is one of the most widely cultivated drought- and high-temperature tolerant C4 cereals, being grown for forage, grain and stover under dryland, rainfed and irrigated conditions in drought-prone regions of the arid and semi-arid tropics and sub-tropics, and as a mulch in conservation tillage production systems in the humid and sub-humid tropics. It is especially important as a staple food grain, and source of feed and fodder for livestock, in hot, dry marginal agricultural production environments of Africa and South Asia that are home to hundreds of millions of the world’s poorest farmers [1]. Besides soil infertility, drought is the most important abiotic constraint to production of this crop, and is a major contributor to the instability of its grain and fodder yields.

The first molecular marker-based genetic linkage map of pearl millet, comprised largely of RFLP loci supplemented by a few isozyme loci, was reported by Liu *et al.*[2]. In subsequent years, the linkage map was expanded with SSR markers [3], and more recently DArT markers [4], and its complex relationships with the foxtail millet and rice genomes were established [5]. Quantitative trait loci (QTLs) for disease resistance [6-10], drought tolerance [11-13], components of drought adaptation [14], flowering time, grain and stover yield [15], and ruminant nutritional quality of straw [16,17] have been mapped, and effective marker-assisted selection for several of these traits has been demonstrated [18,19]. These tools have already been applied for marker-assisted backcross improvement of downy mildew resistance of elite hybrid parental lines, which culminated in the 2005 release in India of pearl millet hybrid “HHB 67 Improved”, which was the first public-bred product of DNA-marker-assisted selection to be released for cultivation in that country [19,20].

SSR markers are one of the best options available for foreground selection in marker-assisted backcrossing programs because they are hyper-variable, multi-allelic, often co-dominant, highly reproducible, and readily multiplexed. They are also ideal for anchoring molecular linkage maps [21] that can be more highly saturated with DArT [4], SNP [22-24], or genotyping-by-sequencing [25] markers. EST-SSRs are of particular interest for linkage map alignments, as they are readily transferable to other pedigrees [26-29] and may functionally determine observed trait variation. To date approximately 150 functional SSR primer pairs have been published for use in pearl millet [3,30-36], in addition to SSCP-SNP [22], DArT [4], CISP and SNP [23,24] markers. However, much larger numbers of markers are required for their more effective application in plant breeding. Further, almost all existing pearl millet molecular markers cluster in regions proximal to the centromeres of the seven linkage groups, with very few loci mapping to distal regions of the chromosomes [3,5,10,12]. There is an urgent need to identify larger numbers of co-dominant polymorphic markers mapping to these distal regions of the pearl millet chromosomes, which are expected to contain the vast majority of genes and gene-associated regulatory sequences.

The limited amount of sequence information in pearl millet has limited progress in gene discovery and characterization, global transcript profiling, probe design for development of gene arrays, and generation of molecular markers and their application in crop improvement programs.

Interestingly, Next Generation Sequencing (NGS) technologies are proving useful for rapidly and efficiently developing genomic resources of minor crop species. In case of under-resourced crop species, where appropriate or adequate sequence data were not yet available, one strategy has been to sequence cDNAs with NGS technologies and then align these sequences with transcript data of that species, if available, with transcript data of any related major/model crop species [37], or with the aligned genome sequences of such model species [38,39]. Combining gene-based markers together with previously available marker systems will greatly assist in filling the gaps in the existing pearl millet linkage maps [24], reducing linkage drag associated with marker-assisted selection, and increasing the speed and efficiency of subsequent QTL introgression programs.

While traditionally a genetic linkage map has been generated from a single population, recent efforts to create maps from multiple populations, referred to as consensus maps, have gained much interest in the scientific and breeding community. Integration of mapping data from individual maps into one consensus map has been reported in forage [40] and cereal species [41-43], including pearl millet [3], and aims to determine the relative positions of transferable markers in order to compare candidate gene and QTL locations across a broad range of genetic backgrounds.

During the process of developing EST resources from drought-stressed leaf and root tissues of selfed progenies from single-plant selections of two elite inbred genotypes differing in terminal drought tolerance (ICMB 841-P3 and 863B-P2), that are also parents of a mapping population [12,13], a contiguous segmental substitution line set [44], and several different QTL introgression line sets, we took the opportunity to develop new EST-based SSR markers. These EST-SSRs, along with other PCR-compatible markers, were then mapped using four pearl millet RIL mapping populations. Subsequently, a consensus map that integrates data from these four linkage maps was constructed.

## Results

### Sequence data assembly

Four cDNA samples synthesized from four total RNA samples [1) leaf RNA from ICMB 841-P3, 2) root RNA from ICMB 841-P3, 3) leaf RNA from 863B-P2, and 4) root RNA from 863B-P2] derived from drought-stressed leaf and root tissues of ICMB 841-P3 and 863B-P2 were sent to the J. Craig Venter Institute (JCVI, USA) in November 2008 for sequencing and assembly using FLX/454 sequencing technology. A single full-plate run on the FLX/454 sequencing machine generated approximately 400 K reads with an average read length of 250–400 bp with the technology available in March 2009 [45]. The four half-plate runs of the normalized pearl millet cDNA libraries on a FLX/454 sequencer generated an average of 184 K reads per half-plate-run while the average read length was 205 bp. The raw ESTs were cleaned of rRNA, vector, ligator and poor quality sequences, which resulted in a reduction in the average number of reads to 99 K per half-plate run, but an increase in the average read length to 224 bp. Cleaned ESTs from the four samples were assembled together using the PLANTTA pipeline at JCVI (see Materials and Methods). This resulted in a total of 34,270 contigs and 78,594 singletons, i.e. a total of 112,864 tentative unique sequences (TUSs) with an average read length of 240 bp. Further, 5,800 putative SNPs (Additional file 1) were identified in 2,146 contigs that were formed from reads derived from these two inbreds. The remaining 32,124 contigs were either formed from sequence reads from a single genotype, or were formed from sequence reads from the two genotypes but were monomorphic with regard to putative SNPs when checked *in silico*.

### Development of EST-SSR markers

All TUSs (112,864) from the PLANTTA pipeline were searched for Class I SSRs [46] using the MIcroSAtellite (MISA) program (http://pgrc.ipk-gatersleben.de/misa/) and 502 Class I SSRs were identified in 499 TUSs. Nineteen TUSs matching to previously published pearl millet SSR markers, based on BLAST search, were removed. The remaining 480 TUSs containing Class I SSRs were analyzed using the CAP3 program. This yielded 341 non-redundant sequences, which were used for primer design with the Primer3 program [47]. These EST-SSR primer pairs were given the prefix name IPES (ICRISAT Pearl millet EST Stress), but only 211 (IPES0001 to IPES0203 and IPES0229 to IPES0236) primer pairs could be designed. An additional 25 primer pairs (IPES0204 to IPES0228) were designed separately from the TUSs resulting from further CAP3 assemblies of cleaned FLX/454 ESTs prepared at ICRISAT-Patancheru. In all, 236 non-redundant EST-SSR primer pairs were designed, of which 212 EST-SSR primer pairs were expected to detect class I SSR loci (*IPES 0001* to *Xipes0203*, *Xipes0226,* and *Xipes0229* to *Xipes0236*) and the remaining 14 were expected to detect class II SSR loci (*Xipes0204* to *Xipes0228*). The forward and reverse primer sequences of these newly developed IPES-series EST-SSRs are given in Additional file 2.

### Linkage mapping and component maps of the four recombinant inbred populations (RIPs)

Among the 236 IPES primer pairs tested, 139 produced amplification products, out of which 119 were polymorphic among parents of at least one of the four RIPs. Apart from the newly developed EST-SSR primer pairs of the IPES series, previously published EST-SSR primer pairs (ICMP series) developed by Senthilvel *et al.*[34], genomic SSR primer pairs (PSMP series) developed by Qi *et al.*[3,31] and Allouis *et al.*[30], genomic SSR primer pairs (CTM series) developed by Budak *et al.*[32], and several STS primer pairs previously developed at John Innes Centre, UK (unpublished) were also assessed for polymorphism detection between the parents of these four RIPs. Among them, 125 primer pairs amplified. The polymorphic markers for each of the RIPs were surveyed on the respective recombinant inbred line (RIL) progeny sets and then mapped using GMendel 3.0 [48], Mapmaker 3.0 [49,50] and RECORD [51]. Map construction was performed for each RIP separately, but as the mapping was being done simultaneously for all four RIPs, we could identify a few of the unlinked groups for one RIP as being sub-groups of larger linkage groups detected on one or more of the other three RIPs. A total of 171 primer pairs, including 99 IPES, 17 ICMP, 47 PMSP, 6 CTM and 2 PSMP(STS) primer pairs, detected polymorphic loci mapped on one or more of the four F_7_ RIPs. The details of the individual maps (Table 1 and Table 2) for each of the four RIPs are:

**Table 1:** Markers mapped among the four individual maps and consensus map

**Maps**	**Marker series**					**Total**	**Distorted markers significant at 1% LOS**
	***Xctm***	***Xicmp***	***Xipes***	***Xpsmp***	***Xpsmp(sts)***		
RIP A (ICMB 841-P3 × 863B-P2)	3	9	64	26	2	104	35 (34%)
RIP B (H 77/833-2 × PRLT 2/89-33	4	11	48	15	0	78	27 (35%)
RIP C (81B-P6 × ICMP 451-P8)	2	1	42	19	0	64	24 (38%)
RIP D (PT 732B-P2 × P1449-2-P1)	0	3	40	16	0	59	18 (31%)
Consensus map	6	17	97	47	2	169	-

**Table 2:** Details of the four individual maps and consensus map

	**RIP A (ICMB 841-P3 × 863B-P2)**		**RIP B (H 77/833-2 × PRLT 2/89-33)**		**RIP C (81B-P6 × ICMP 451-P8)**		**RIP D (PT 732B-P2 × P1449-2-P1)**		**Consensus map**	
**Linkage groups**	**No. of markers**	**Map length (cM)**	**No. of markers**	**Map length (cM)**	**No. of markers**	**Map length (cM)**	**No. of markers**	**Map length (cM)**	**No. of markers**	**Map length (cM)**
LG1 or LG1a	18	130	17	123	13	76	6	29	29	147
LG1b							2	0.3		
LG2	18	104	14	139	10	108	4	6	30	193
LG3	8	44	9	102	7	54	8	34	17	94
LG4	12	49	6	68	6	21	9	69	17	87
LG5	13	86	8	84	10	117	9	48	22	134
LG6 or LG6a	20	98	14	86	7	10	4	9	32	113
LG6b					4	8	2	3		
LG6c					2	3	2	2		
LG7	15	104	10	88	5	31	11	74	27	130
LGA (unlinked)	-	-	-	-	-	-	2	2	-	-
Total	**104**	**615**	**78**	**690**	**64**	**428**	**59**	**276**	**174**	**898**

RIP A (= ICMB 841-P3 × 863B-P2)

A total of 64 *Xipes*, 9 *Xicmp*, 26 *Xpsmp*, 3 *Xctm* and 2 *Xpsmp(sts)* marker loci were mapped on 7 linkage groups, having a total map length of 615 cM, an average length of 88 cM per linkage group, and an average inter-marker distance of 6 cM. The linkage maps of RIP A are given in Additional file 3.

RIP B (= H 77/833-2 × PRLT 2/89-33)

A total of 48 *Xipes,* 11 *Xicmp*, 15 *Xpsmp* and 4 *Xctm* marker loci were mapped on 7 linkage groups, having a total map length of 690 cM, an average length of 99 cM per linkage group, and an average inter-marker distance of 9 cM. The linkage maps of RIP B are given in Additional file 4.

RIP C (= 81B-P6 × ICMP 451-P8)

A total of 42 *Xipes,* 1 *Xicmp*, 19 *Xpsmp* and 2 *Xctm* marker loci were mapped on 7 linkage groups, having a total map length of 428 cM, an average length of 61 cM per linkage group, and an average inter-marker distance of 7 cM. Linkage group 6 (LG 6) was obtained as 3 sub-groups containing 7, 4, and 2 markers, with map lengths of 10, 8, and 3 cM, respectively. The linkage maps of RIP C are given in Additional file 5.

RIP D (= PT 732B-P2 × P1449-2-P1)

A total of 40 *Xipes,* 3 *Xicmp* and 16 *Xpsmp* marker loci were mapped on the expected 7 linkage groups (LG1 through LG7) and one unlinked group (LGA) with a total length of only 276 cM. The average length for the 7 expected linkage groups was 29 cM, and their average inter-marker distance was 5 cM. LG1 was obtained as 2 sub-groups, LG1a and LG1b, with 6 and 2 markers, and map lengths of 29 and 0.3 cM, respectively. LG6 was obtained as 3 sub-groups, LG6a, LG6b and LG6c, with 4, 2 and 2 markers, and map lengths of 9, 3 and 2 cM, respectively. The linkage maps of RIP D are given in Additional file 6.

Segregation distortion (Table 1) of mapped markers ranged from 31% (RIP D) to 38% (RIP C). The newly developed *Xipes*-series markers showed distortion in the range of 25% (RIP D) to 36% (RIP C). This marker distortion favored alleles of female or male parents, depending on the RIP: “female parent ICMB 841-P2” (91%), “male parent PRLT 2/89-33” (93%), “male parent ICMP 451-P8” (58%) and “female parent PT 723B-P2” (78%), for RIPs A, B, C and D, respectively.

A comparative map was developed using MapChart 2.2 [52] with the maps of the four RIPs (Additional file 7). There were five primer pairs that detected at least two polymorphic loci:

•IPES0027, which detected *Xipes0027.1* on LG6 in RIP B and *Xipes0027.2* on LG2 in RIP C and RIP D;

•IPES0152, which detected *Xipes0152.1* on LG2 in RIP B and *Xipes0152.2* on LG5 in RIP A, RIP C and RIP D;

•PSMP2229, which detected *Xpsmp2229.3* on LG3 in RIP C and *Xpsmp2229.1* on LG5 in RIP A (and is previously reported to detect *Xpsmp2229.2* on LG7 as well [3]);

•IPES0220, which detected *Xipes0220.1* on LG3 in RIP B and *Xipes0220.2* on LG5 in RIP C; and,

•PSMP2081, which detected *Xpsmp2081.1* on LG4 in RIP A and RIP D, and *Xpsmp2081.2* on LG6 in RIP B.

**Figure 1:**
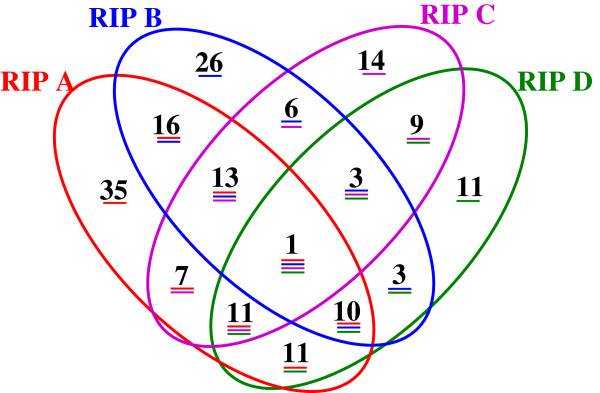
**Venn diagram showing marker overlap across four RIL mapping populations.** A four-way Venn diagram illustrating all unique, two-way, three-way and four-way sets of shared markers. The mapping populations are abbreviated as in the text: RIP A = ICMB 841-P3 × 863B-P2; RIP B = H 77/833-2 × PRLT 2/89-33; RIP C = 81B-P6 × ICMP 451-P8; RIP D = PT 732B-P2 × P1449-2-P1.

The numbers of common markers across the four RIPs were identified and displayed in a Venn diagram (Figure 1). In all, 176 marker loci were mapped among the four RIPs. Among these, 90 marker loci were shared between sets of any two or three or four RIPs. There was only one polymorphic locus (*Xipes0093* on LG2) shared across all four RIPs. One additional primer pair (IPES0152) detected polymorphic loci across all four RIPs (one locus on LG2 of RIP B, and a second locus on LG5 of RIP A, RIP C and RIP D). Similarly, 37 marker loci were shared between sets of 3 RIPs, 52 marker loci were shared between pairs of RIPs, and 86 marker loci were unique to one or the other of the four RIPs. The details of shared polymorphic loci are provided in Additional file 8.

Primer pairs previously reported to detect more than one polymorphic locus, which only detected a single mapped locus in this study, included PSMP2232 [3], PSMP2263 [34] and PSMP2270 [34]. On average, 13 shared marker loci were present on each linkage group. This substantial number of shared marker loci facilitated the production of a consensus map.

### Consensus map

**Figure 2:**
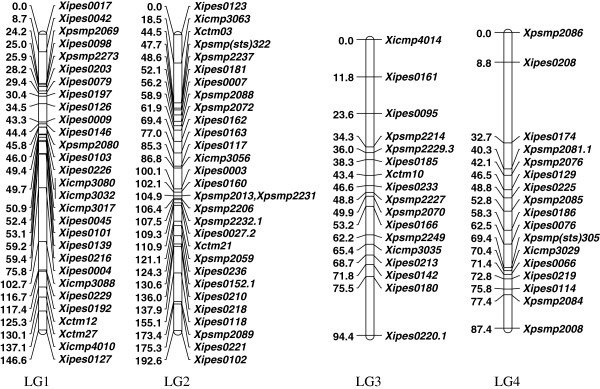
**Pearl millet SSR marker consensus map for LG1 through LG4 based on four RIL mapping populations.** Linkage distances are given in Haldane cM on the left side of each bar and the marker names are given on the right side of each bar.

**Figure 3:**
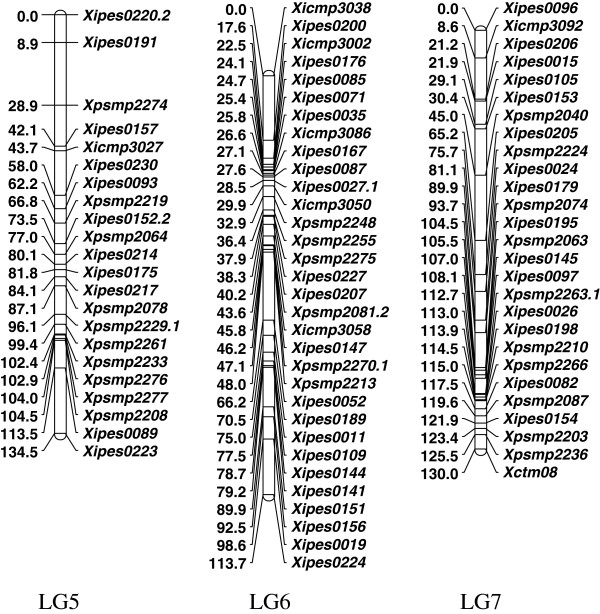
**Pearl millet SSR marker consensus map for LG5 through LG7 based on four RIL mapping populations.** Linkage distances are given in Haldane cM on the left side of each bar and the marker names are given on the right side of each bar.

The four maps were integrated using MergeMap [53] to form a consensus map comprised of the expected 7 linkage groups, containing 174 marker loci from 169 markers with a total map length of 899 cM (Figures 2 and 3, and Additional file 7). Another two markers, namely *Xipes0014* and *Xipes0110* belonging to LGA of RIP D, were not integrated in the consensus map as they were not associated with any of the seven expected linkage groups. The map lengths of linkage groups in the consensus map were 147, 193, 94, 87, 134, 113 and 130 cM for LG1, LG2, LG3, LG4, LG5, LG6 and LG7 with 29, 30, 17, 17, 22, 32 and 27 marker loci, respectively (Table 1 and Table 2).

### Syntenic relationships of pearl millet with sequenced grasses

BLASTn results for mapped pearl millet gene-based SSR markers (EST-SSR markers), for each chromosome of each of the five available sequenced cereal genomes, were combined with marker locus orders from the pearl millet linkage analysis to identify likely macro-level syntenic relationships. Possible segmentally syntenic relationships of the pearl millet linkage groups (Pg_1 = LG1, Pg_2 = LG2 , Pg_3 = LG3, Pg_4 = LG4, Pg_5 = LG5, Pg_6 = LG6, Pg_7 = LG7 and Pg_A = LGA) were identified (Table 3 and Additional file 9) for each of the following grasses: chromosomes of foxtail millet (Si_1 to Si_9), chromosomes of rice (Os_01 to Os_12), chromosomes of sorghum (SBI-01 to SBI-10), chromosomes of maize (Zm_01 to Zm_10) and chromosomes of *Brachypodium* (Bd_1 to Bd_5). The BLASTn top hits (*e*-value ≤ 1E-10) used for this were for the full-length sequences from which the pearl millet EST-SSR primer pairs (IPES and ICMP series) were designed and whose corresponding loci were mapped. Pearl millet linkage groups Pg_1, Pg_2, Pg_3, Pg_4, Pg_5, Pg_6, Pg_7 and Pg_A have 21/29 (21 out of 29), 17/30, 6/11, 9/17, 9/22, 16/32, 10/27 and 1/2 marker loci, respectively, that show likely syntenic relationships with these cereals. The orders of the loci of the gene-based markers (*Xipes* series and *Xicmp* series) on the pearl millet linkage groups and the corresponding regions of chromosomal segments of the five cereal genomes are reasonably well conserved for much of the length of the pearl millet linkage groups, despite the greater degree of genomic restructuring that appears to have occurred in this species compared to better-studied grasses and which is thought responsible for the relatively large number of apparent segmental translocation, inversion and insertion events that would be required to construct the pearl millet chromosome architecture from that of the putative ancestoral grass [5]. For example the order of five marker loci (*Xipes0071*, *Xipes0085*, *Xipes0176*, *Xicmp3002* and *Xipes0200*) on pearl millet linkage group 6 and its syntenic regions is highly conserved in foxtail millet, its closest relative, and less so in more distantly related grasses (Additional file 9). Out of the 119 EST-SSR marker loci (17 *Xicmp* series loci and 102 *Xipes* series loci) mapped across one or more of the four pearl millet RIPs, good BLAST hits (*e*-value ≤ 1E-10) were detected with at least one of these five cereal genomes for 89 loci. Among these 119 loci, 29.4% (35 loci), 74% (88 loci), 48.7% (58 loci), 40.3% (48 loci) and 31.9% (38 loci) had detectable relationships with the portions of the rice, foxtail millet, sorghum, maize and *Brachypodium* genomes, respectively. This suggests that pearl millet is most closely related to foxtail millet, followed by sorghum, maize, rice and *Brachypodium* in decreasing order, which is in agreement with current understanding of grass evolution [54-56].

**Table 3:** Numbers of mapped pearl millet gene-based markers, by linkage group, exhibiting significant relationships with loci on chromosomes of five sequenced grasses; chromosomes indicated in bold font show best evidence for synteny with the corresponding pearl millet linkage groups

**Sno**	***Oryza sativa (Rice)***	***Setaria italica *(Foxtail millet)**	***Pennisetum glaucum *(Pearl millet)**	***Sorghum bicolor *(Sorghum)**	***Zea mays *(Maize)**	***Brachypodium distachyon***
1	Os_12(1)	**Si_7(2)**	Pg_1(21)	**SBI-08(2)**	**Zm_10(6)**	Bd_4(1)
	Os_10(1)	Si_1(1)		SBI-04(1)	Zm_08(1)	Bd_3(1)
	**Os_05(5)**	**Si_3(11)**		**SBI-09(9)**	**Zm_06(3)**	**Bd_2(7)**
		Si_6(2)		SBI-01(1)	Zm_02(1)	
		**Si_8(4)**				
2	**Os_03(2)**	**Si_9(10)**	Pg_2(17)	**SBI-01(6)**	**Zm_01(4)**	**Bd_1(2)**
	**Os_10(2)**	Si_4(2)		SBI-10(2)	Zm_06(1)	Bd_5(1)
	Os_02(1)	**Si_1(4)**		**SBI-04(3)**	Zm_09(1)	**Bd_3(4)**
		Si_2(1)			**Zm_05(4)**	
3	**Os_02(3)**	**Si_1(4)**	Pg_3(6)	**SBI-04(4)**	Zm_05(1)	Bd_1(1)
	Os_01(1)	Si_7(1)		SBI-03(1)	**Zm_04(2)**	**Bd_3(2)**
		Si_3(1)			Zm_03(1)	Bd_2(1)
4	Os_01(1)	Si_5(1)	Pg_4(9)	SBI-09(1)	**Zm_01(3)**	Bd_2(1)
	**Os_08(4)**	Si_3(1)		SBI-08(1)	Zm_06(1)	**Bd_3(3)**
		**Si_6(6)**		**SBI-07(3)**	Zm_10(1)	
		Si_9(1)		SBI-05(1)	Zm_02(1)	
5	**Os_03(5)**	Si_3(1)	Pg_5(9)	**SBI-01(5)**	**Zm_01(2)**	**Bd_1(5)**
		**Si_4(3)**			**Zm_09(2)**	
		**Si_9(5)**				
6	Os_04(1)	Si_7(1)	Pg_6(16)	SBI-06(1)	Zm_06(1)	Bd_5(2)
	Os_11(1)	Si_1(1)		SBI-05(1)	Zm_04(1)	Bd_4(1)
	**Os_01(4)**	**Si_5(14)**		SBI-02(1)	Zm_02(1)	Bd_2(2)
				**SBI-03(7)**	Zm_03(5)	
7	Os_09(1)	Si_9(1)	Pg_7(10)	SBI-09(1)	Zm_02(1)	Bd_4(1)
	Os_07(1)	**Si_2(9)**		**SBI-02(6)**	**Zm_07(3)**	**Bd_1(2)**
8	**Os_02(1)**	**Si_4(1)**	**Pg_A(1)**	**SBI-04(1)**	**Zm_05(1)**	**Bd_1(1)**
Total	(35)	(88)	(89)	(58)	(48)	(38)

Linkage groups of pearl millet represented as Pg_1 = LG1, Pg_2 = LG2, Pg_3 = LG3, Pg_4 = LG4, Pg_5 = LG5, Pg_6 = LG6, Pg_7 = LG7 and Pg_A = LGA, Chromosomes of foxtail millet named as Si_1 to Si_9, chromosomes of rice as Os_01 to Os_12, chromosomes of sorghum as SBI-01 to SBI-10, chromosomes of maize as Zm_01 to Zm_10 and chromosomes of *Brachypodium* as Bd_1 to Bd_5. The numbers in the brackets indicate the number of pearl millet TUSs containing mapped EST-SSR markers that had significant BLAST hits (*e*-value ≤ 1E-10) on the chromosome sequences of other grasses when BLAST search was done separately for each grass genome.

## Discussion

In this study we have identified high quality polymorphic EST-SSRs and these have enriched the marker resources of generally marker-poor pearl millet. The newly developed EST-SSRs will be useful in genetic diversity assessment, genome mapping, QTL mapping, association mapping and marker-assisted breeding experiments.

Initially, 236 EST-SSR primer pairs were designed from the FLX/454 sequence data, and have been tested for amplification and ability to detect polymorphism using template DNA from parental inbreds of four pearl millet RIL mapping populations. The main criteria used to select the primer pairs for genetic mapping were reproducibility, ability to produce single and/or well-defined scorable peaks with an automated florescence-based genotyping system (ABI3730xl manufactured by Applied Biosystems, USA), large repeat length (class I SSRs), amenable for automation, product size in the range of 100 to 500 bp, and detecting scorable polymorphism for one of the four parental pairs tested. These stringent criteria reduced the number of primer pairs in the working set to 99. Trinucleotide repeat markers were more highly polymorphic (38%) than the dinucleotide (16%), tetranucleotide (18%) and pentanucleotide (19%) repeat-based markers, as observed previously in pearl millet [33,34].

RIP A had the highest number of polymorphic marker loci (104), while RIP D had the lowest number of polymorphic loci (59). RIP B had the greatest total map length; however, this total map distance was inflated by markers loosely mapping to the distal ends of several linkage groups. It was also noted that the distribution of markers in a particular LG were not uniform across RIPs. For example, 18 markers mapped to LG2 of RIP A, whereas just four markers mapped to LG2 of RIP D.

Segregation distortion occurred uniformly across genomic regions, with the specific regions involved varying from RIP to RIP. Segregation distortion is a common phenomenon in pearl millet and has been reported in essentially all earlier mapping studies of this cross-pollinated species [2-4,7-17,24,57-59]. Generally, segregating populations have differential levels of segregation distortion, but RILs exhibit stronger distortion of marker segregation than do earlier-generation mapping populations. It has been suggested that involuntary selection against a few genomic regions during generation of the RILs, or incompatibility between genomic regions contributed by the different parents [60], contribute to the higher levels of segregation distortion observed in RIPs.

The bootstrap histogram (not shown) of individual LGs of the four RIPs revealed that the order of the markers were well conserved and all of the single-copy markers in all LGs showed unique positions except those that are very closely linked (where the small sizes of the RIPs used in this study resulted in differences in marker order that are likely to be artifacts). Even these sets of closely-linked markers shared their position with markers in nearby regions. The unique positions of these markers, in spite of the observed segregation distortion, is indicative of the stability of the pearl millet LGs, provided that there are no differences in chromosome structure such as those reported in the first RFLP-based pearl millet linkage map [2].

A total of 171 markers mapped to 176 loci (Table 4) on the expected 7 linkage groups and an unlinked group (LGA) of the four RIPs, and these markers were relatively uniformly distributed (at least across regions of the nuclear genome proximal to the centromeres of the seven pearl millet chromosome pairs). The newly developed *Xipes*-series EST-SSRs have been positioned relative to previously published SSR markers and genetic linkage maps of pearl millet. The map order of marker loci in the four RIPs were generally consistent with previously published SSR-based maps of pearl millet [3,14,34]. RIP D had an average inter-marker distance of 4.7 cM followed by RIP A with 5.9 cM, RIP C with 6.7 cM, and RIP B with 8.8 cM. This optimal inter-marker distance, and the uniform coverage across the nuclear genome will provide greater opportunities to locate QTLs that have not been identified so far and will be particularly useful for the identification of recombination events adjacent to regions targeted for introgression in marker-assisted backcrossing programs, which are required to minimize negative linkage drag that could result from introgression of large donor segments flanking each introgression target [61-67].

**Table 4:** Summary of markers and marker loci

**Title**	**No. of primer pairs**	**No. of marker loci**
Total mapped in four crosses	171	176
(*Xctm* + *Xicmp* + *Xipes* + *Xpsmp* + *Xpsmp*(*sts*))	(6+17+99+47+2)	(6+17+102+49+2)
Total mapped in consensus map	169	174
(*Xctm* + *Xicmp* + *Xipes* + *Xpsmp* + *Xpsmp*(*sts*))	(6+17+97+47+2)	(6+17+100+49+2)
Total significant BLAST hits (*e*-value ≤ 1E-10) detected on rice, foxtail millet, sorghum, maize or *Brachypodium* genomes	87	89
(*Xicmp* + *Xipes*)	(14+73)	(14+75)

The presence of gaps in the distal regions of a few linkage groups was due to the forceful assignment of markers to the distal ends of these groups using MapMaker 3.0. However care was taken while assigning these markers to individual linkage groups by looking at their map positions in other RIPs. *Xipes0221* was assigned to the distal region of LG2 in RIP C after considering its position in this region of LG2 for RIP A. In the same way, a sub-group of markers linked to *Xipes0144* and another sub-group of markers linked to *Xipes0156* were assigned to LG6 for RIP C and RIP D, based on their linkage relationships in RIP A. The presence of gaps in the sub-telomeric regions of these linkage groups is probably due to very high recombination rates in these regions (considered most likely), the presence of marker- or gene-poor regions immediately adjacent to the telomeres of each chromosome arm (considered unlikely), or the absence of markers that can effectively link sub-telomeric and centromeric regions. Similar gaps were reported previously by Devos *et al.*[5], using RFLP probes to establish the syntenic relationships between genetic maps of rice, foxtail millet and pearl millet. However, other marker systems that cover the entire genome (such as DArT and GbS-SNPs, provided that appropriate endonucleases have been used during complexity reduction) need to be mapped in these RIPs to confirm this phenomenon. An attempt has been made in this direction by Supriya *et al.*[4], using 258 DArT and 63 SSR markers to cover the nuclear genome of pearl millet RIP B. That study greatly extended the marker coverage in sub-telomeric regions of all seven pearl millet linkage groups.

In the current study, we have constructed a consensus map or integrated linkage map for pearl millet using MergeMap, which outperforms JOINMAP both in terms of accuracy and running time [53]. This consensus map is simply one of many possible non-conflicting linear representations of the consensus directed acyclic graphs (DAGs). However, the order of mapped loci was generally well conserved between the integrated map and the RIP-specific maps, which indicates that the positions of the loci on the present integrated map can be regarded as a good “consensus map”. Unlike the integrated map published by Qi *et al.*[3], this consensus map, which is mostly based on EST-SSRs (68%) and genomic SSRs (31%), has a more or less uniform distribution of markers across all seven expected pearl millet linkage groups.

The marker positions in the consensus map obtained using MergeMap were verified using the DAG files generated by the MergeMap tool and by using the comparative maps (Additional file 7) generated using MapChart. Locus order was well conserved across all of the linkage groups, except for LG3, which exhibited conflicting marker positions for 4 loci. There were no conflicts in marker positions for LG2 and LG5. LG1 had a conflict for the marker positions of *Xipes0126* and *Xipes0139*; LG3 had such conflicts for *Xipes0142*, *Xipes0180*, *Xipes0213* and *Xipes0095*. On LG3, the markers *Xpsmp2227* and *Xipes0166* mapped adjacent to each other, but their order was inverted when maps of RIP A and RIP C were compared. LG4 had conflicts for two markers, *Xipes0066* and *Xipes0219*, which mapped adjacent to each other without any marker between them. Their positions were inverted when maps of RIP C and RIP D were compared. For LG6, the marker loci *Xpsmp2270* and *Xipes0207* were inverted when maps of RIP A and RIP C were compared with that of RIP B. Finally, LG7 had a conflict for the positions of adjacent markers *Xipes0206* and *Xipes0153*, which have inverted positions when maps of RIP A and RIP D are compared. Five SSR primer pairs detected more than one polymorphic locus, which could be due to translocation events in the genomic regions involving loci detected by primer pairs, or more likely, could be due to the presence of conserved SSRs occurring in multi-gene families or duplicated genomic regions.

The availability of published or draft genome sequences of rice, foxtail millet, sorghum, maize and *Brachypodium* made it possible to perform BLAST searches of the pearl millet EST sequences (from which EST-SSR primer pairs had been designed) against these genomes to identify possible syntenic relationships between the pearl millet linkage map and the aligned genomes of these sequenced grasses (Additional file 9). This allowed us to determine that previously unassigned group LGA of RIP D is likely to be a part of LG3. A more dense or higher resolution map with additional EST-based markers would give a much better picture of the possible syntenic relationships between the genome of pearl millet and those of other sequenced species.

## Conclusion

Linkage maps form a framework for trait mapping and QTL analysis. The newly developed EST-SSR markers (99 IPES series primer pairs), along with primer pairs for previously mapped EST-SSRs and SSRs (70) and STS (2) markers were used to construct separate linkage maps of four F_7_ recombinant inbred populations. These linkage maps were used to construct a consensus map for pearl millet with 169 primer pairs detecting 174 marker loci. The locus order of the present consensus map is highly consistent, and is sufficiently reliable for use as a reference genetic map for pearl millet. Overall, the consensus map developed in the present study contains the largest set of mapped SSRs reported to date in pearl millet, and represents a major consolidation to existing pearl millet genetic mapping information. The suggested syntenic relationships of the pearl millet linkage groups with chromosomes of rice, foxtail millet, sorghum, maize and *Brachypodium* demonstrate that these grasses are closely related. The consensus map, the four RIL populations upon which it is based, and the syntenic relationships between these grasses identified based on the new pearl millet EST-SSRs, will prove to be useful assets in the development of both molecular breeding for pearl millet and for comparative genetics and genomics within the grass family.

## Methods

### Plant materials

Selfed progeny of single-plant selections (previously used as mapping population parents), ICMB 841-P3 and 863B-P2 from a genetically diverse pair of elite pearl millet inbred lines, were used to construct the EST library. ICMB 841 was bred at ICRISAT-Patancheru by pure-line selection for downy mildew resistance in a seed lot of elite maintainer line MS 5141B [68]. It has small seed size, narrow and pubescent leaf blades, thin panicles, thin stems and poor combining ability for terminal drought tolerance. It is the product of an outcross to an unidentified parent that contributed it improved downy mildew resistance compared to its seed parent MS 5141B [69]. 863B was bred at ICRISAT-Patancheru from *Iniadi* landrace material from Togo by selfing and selection [70]. It has large grain size, broad and glabrous leaf blades, thick stems, and thick panicles, superior downy mildew resistance, and good combining ability for terminal drought tolerance and good stover quality (associated, at least in part, with a major gene for partial resistance to blast disease caused by the fungus *Magneporthe grisea*[71]). Mapping populations developed from the cross of these two elite seed parent maintainer lines have been used to map downy mildew resistance [72], terminal drought tolerance [12,13], phenological traits [16], ruminant nutritional value of stover [17,71] and grain concentrations of mineral micronutrients Fe and Zn [73].

### RIL populations

 ICMB 841-P3 × 863B-P2 (RIP A)

This RIL population consists of 106 F_7_ RILs and is segregating for combining ability for terminal drought tolerance, grain and stover yield components, grain and stover quality traits, as well as segregating as inbreds *per se* for host plant resistance to both downy mildew and blast, salinity tolerance, grain density of Zn and Fe, and perhaps tolerance to alkaline soil conditions. Earlier generations of this mapping population have been used to map terminal drought tolerance [12,13] and downy mildew resistance [72], as well as plant height, flowering time, and components of ruminant nutritional value of pearl millet straw [16,17], and grain mineral micronutrient levels [73]. They have also been used previously for addition of EST-SSR markers to the earlier RFLP and STS marker-anchored pearl millet linkage map [34].

H 77/833-2 × PRLT 2/89-33 (RIP B)

H 77/833-2 is tolerant to seedling heat stress and intermittent drought stress, and sensitive to terminal drought stress, whereas PRLT 2/89-33 is sensitive to seedling heat stress and pre-flowering drought stress, but tolerant to terminal drought stress. H 77/833-2 tillers profusely (both basally and nodally), has thin stems, narrow and glabrous leaf blades, small panicle volume, and very small grain size. H 77/833-2 was the male parent of three hybrids (HHB 60, HHB 67 and HHB 68) bred and released from CCS Haryana Agricultural University. In contrast, PRLT 2/89-33 usually produces a single effective culm, has a thick stem, broad and pubescent leaf blades, longer and thicker panicles (hence a larger panicle volume), and moderately large grain size. These parents are genetically diverse, agronomically elite restorer lines, and hence, with the parents of RIP A above form a complementary set of materials useful for mapping a multitude of traits in agronomically elite hybrid backgrounds adapted to a wide range of growing conditions typical for pearl millet in peninsular and northwestern India. This RIP consists of 145 F_7_ RILs segregating for seedling heat-stress tolerance, terminal drought-stress tolerance, grain and stover yield components, and downy mildew resistance. It has recently been used to place additional gene-based markers into the genomic region associated with a major drought tolerance QTL [24], and to map physiological components of this terminal drought tolerance QTL [14]. Earlier generations of this mapping population have been used to map terminal drought tolerance [11], and QTL × E interactions for grain and stover yield components across seven natural dryland and managed (stress or non-stress) moisture environments in India [15], and downy mildew resistance [72].

81B-P6 × ICMP 451-P8 (RIP C)

81B-P6 is semi-dwarf (*d*_2_), with long and narrow pubescent leaf blades (*hl*), limited basal-tillering capacity, and long, thin, short-bristled panicles. It is highly susceptible to rust, maintains male-sterility for the A_1_, A_4_ and A_5_ pearl millet cytoplasmic male-sterility systems, and is a single-plant selection from commercially important maintainer line 81B = ICMB 1 [74]. 81B is the product of an outcross [69] with an unknown downy mildew resistance source that was made during the course of a mutation program intended to enhance downy mildew resistance of elite, dwarf hybrid seed parent maintainer line Tift 23D_2_B_1_. ICMP 451-P8 is tall with glabrous, brown-splotched leaf blades and has long-bristled semi-compact panicles, amber-grey colored globular seeds and is slow-rusting. It is one of three single-plant selections from elite pollinator inbred ICMP 451 [75] that have been used as mapping population parental lines – the other two populations were reported by Busso *et al.*[57] and Breese *et al.*[9]. This RIP consists of 170 F_7_ RILs and is segregating for plant height, leaf blade pubescence, long panicle bristles, grain and stover yield components, host plant resistance to rust and downy mildew, and fertility restoration/sterility maintenance for the A_1_ and A_4_ cytoplasmic genetic male-sterility systems. The inbreds crossed to produce this RIP were parents of a widely cultivated, full-season, dual-purpose hybrid released in India in 1986 (ICMH 451 = MH 451), and grown on over 1 m ha annually at the peak of its adoption, before ultimately succumbing to downy mildew in the late 1990s.

PT 732B-P2 × P1449-2-P1 (RIP D)

PT 732B-P2 is agronomically elite, *d*_*2*_ dwarf, and photoperiod-sensitive and is a single-plant selection derived from agronomically elite seed parent maintainer line PT 732B, bred at Tamil Nadu Agricultural University [76]. It is reported to be derived from a “spontaneous dwarf mutant” that was found in a landrace accession from Andhra Pradesh. P1449-2-P1 is late-flowering, tall, and downy mildew and rust resistant. It is single-plant selection from a partially inbred germplasm accession (IP 5853) that exhibited relatively stable downy mildew resistance in multi-locational international nurseries conducted across years and locations in South Asia and sub-Saharan Africa [77]. This RIP consists of 130 F_7_ RILs and is segregating for plant height, downy mildew resistance and rust resistance.

### Drought stress treatments using standard dry-down conditions

Standard dry-down experiment [78] conditions (a fully-irrigated non-stress control paired with slow-onset stress treatments initiated at the beginning of emergence of the main stem panicle from the boot leaf sheath) were used to impose drought stress. The experiment was setup in pots on the floor of a greenhouse maintained at approximately 35°C/25°C, and irrigated as needed until stem elongation of each genotype was initiated. Daily watering continued until 10% of plants of a particular genotype had reached the boot stage of growth. All pots of that genotype were then watered thoroughly to saturate the soil, and allowed to drain overnight. The following morning, which was counted as the first day of stress treatment, each pot was enclosed in a plastic bag to prevent soil evaporation, with an opening for application of irrigation water. On each subsequent day each pot was weighed to measure transpirational water losses, which were replaced according to the following protocol: for plants assigned to the stress treatment, transpirational water losses in excess of 100 ml per day were added back; for plants assigned to the non-stress treatment, transpirational water losses were almost fully replaced to maintain soil moisture at about 80% field capacity. This allowed the stress imposition to progress slowly, as is the case in the field. The stress treatment continued until transpirational water losses of the stressed plants dropped to 20% normalized transpiration ratio (NTR) [78].

### RNA extraction, FLX/454-sequencing and assembly

The drought stressed leaf and root tissues of each of the two inbred genotypes were sampled at 4 days after initiation of the stress treatment, 70% NTR, 40% NTR and at 20% NTR, separately. RNA was extracted using the ‘acid phenol method’ [79]. Finally four pools of total RNA were prepared from the stressed tissues: (1) leaf RNA from ICMB 841-P3, (2) root RNA from ICMB 841-P3, (3) leaf RNA from 863B-P2, and (4) root RNA from 863B-P2. Synthesis of cDNA was done according to the Super SMART™ PCR cDNA synthesis protocol (Clontech Laboratories, Inc., Mountain View, CA, USA). The four cDNA samples, each of approximately 5 μg, were sent to the J. Craig Venter Institute (JCVI, USA), for FLX/454-sequencing and assembly. For each of the four samples, one half-plate run (half of the PicoTiterPlate) was performed on the FLX/454 sequencing machine. The resulting ESTs were cleaned of rRNA, vector, ligator and poor quality sequences using SeqClean (http://compbio.dfci.harvard.edu/tgi/software/) and assembled using the Plant Transcript Assemblies (PLANTTA) pipeline [80], using the TGICL assembler [81] with the following parameters: retention requiring a 50 bp minimum match, 95% minimum identity in the overlap region and 20 bp maximum unmatched overhangs. The contigs and singletons resulting from the PLANTTA assembly are available at the following links, respectively: http://gcpcr.grinfo.net/files/cr_files/gcpcr_file1016.xlsx and http://gcpcr.grinfo.net/files/cr_files/gcpcr_file1017.xlsx.

The CAP3 assembly program [82] was used to do a separate assembly using the cleaned FLX/454 ESTs prepared at ICRISAT-Patancheru (data not used except for primer design of a few sequences and not submitted to database). CAP3 assembly default criteria used were: retention required a 40 bp minimum match, 90% minimum identity in the overlap region and 20 bp maximum unmatched overhangs. Putative SNPs were identified in the contigs formed from reads from ICMB 841-P3 and 863B-P2 based on scripts that are part of the PLANTTA pipeline [80]. The minimal requirement for SNP calling is that there must be at least 2 sequences with the same base. These putative SNPs are listed in Additional file 1.

### EST-SSR primer design and polymorphism screening

The EST sequences were scanned using a local version of the MIcroSAtellite (MISA) program (http://pgrc.ipk-gatersleben.de/misa/) to identify class I SSRs with the parameters: (i) unit size / minimum number of repeats: (2/10) (3/7) (4/5) (5/4) (6/4) and (ii) maximal number of bases interrupting 2 SSRs in a compound microsatellite = 100. The SSR-containing sequences were used to develop EST-SSR primer pairs with the Primer3 program (link to details of EST-SSR primer pairs developed: http://gcpcr.grinfo.net/files/cr_files/gcpcr_file1021.xlsx). The forward primers were synthesized with an m13-sequence (5′CACGACGTTGTAAAACGAC3′) tail on the 5′ end. PCR was performed in a 5 μl reaction volume containing 5 ng genomic DNA template, 0.2 picomole of m13-tailed forward primer, 1 picomole of reverse primer, 1 picomole of dye-labeled m13 primer, 0.5 μl of 2 mM dNTPs, 0.1 U *Taq* DNA polymerase and 0.5 μl of 10X PCR buffer in a Gen-Amp PCR system 9700® thermocycler (Applied Biosystems, USA). PCR conditions were as follows: denaturation at 94°C for 5 min, followed by 10 cycles of denaturation at 94°C for 15 s, annealing at 61°C to 51°C (touch-down cycles) for 30 s, and extension at 72°C for 30 s, followed by 40 cycles of denaturation at 94°C for 10 s, annealing at 54°C for 30 s, and extension at 72°C for 30 s, followed by final extension at 72°C for 20 min. PCR amplification was checked on 1.2% agarose gels and PCR products were separated by capillary electrophoresis on an ABI3730xl sequencer and their sizes were determined using GeneMapper v4.0 software (Applied Biosystems, USA). The primer pairs were screened for their ability to detect polymorphism between parental pairs of the four RIL populations.

### Individual and consensus map construction

In addition to the polymorphic EST-SSRs (*Xipes* series) developed in this study, EST-SSRs (*Xicmp* series), genomic SSRs (*Xpsmp* and *Xctm* series), and STS (*Xpsmp*(*sts*)) markers were mapped using the four RIL populations. GMendel 3.0 was used to create linkage groups with LOD ≥3. The final order of the linkage groups were tested and verified by 25,000 bootstrap iterations. Some of the unlinked markers were assigned to the distal ends of the linkage groups by using “TRY” and “BUILD” commands in MapMaker 3.0. The loci in each linkage group were then ordered using RECORD and the Haldane mapping function was used to calculate inter-marker distances. The graphical representations of individual linkage maps for each mapping population and the correspondence of common markers across populations, were drawn using MapChart. An integrated map combining the respective linkage groups of the four component maps was created using MergeMap. MergeMap calculates a consensus marker order based on the marker order from individual maps. First, a set of DAGs are generated from the individual maps. These DAGs are used as input by the MergeMap to generate a set of consensus DAGs. Each of the consensus DAGs is consistent with all (or nearly all) of the markers in the individual input maps. Each of the consensus DAGs is linearized by MergeMap using a mean distance approximation. The consensus map coordinates are then normalized to the arithmetic mean cM distance for each linkage group from the four individual maps. The consensus map output files from MergeMap were visualized by Graphviz (http://www.graphviz.org/) and the linearized consensus map for each linkage group was visualized by MapChart.

### Identification of synteny

Syntenic relationship of the pearl millet linkage groups were identified with the following grass genome sequences: chromosomes of rice (genome release version “IRGSP Release Build 5.0 Pseudomolecules of Rice Genome”) [83], foxtail millet (foxtail millet genome release by Beijing Genomics Institute in 2012) [84], sorghum (genome release version “JGI Sbi/SBGDB161 (SEPT2007) - Release1”) [85], maize (genome release version “AGPv1, 2009-03-20”) [86] and *Brachypodium* (genome release version “Brachypodium v1.0”) [87]. BLAST search of the full-length pearl millet EST sequences, from which primer pairs (IPES and ICMP series) for mapped EST-SSRs had been developed, was done separately against each of the five genomes mentioned above. The top BLASTn hits on each of the five genomes with *e*-values ≤1E-10 were considered as potentially syntenic for the respective marker loci on pearl millet. The consensus map of pearl millet developed in this study by merging the four linkage maps was combined with the BLAST results to identify the syntenic relationships between the pearl millet linkage groups and the chromosomes of these five grass genomes. The regions of chromosomes of these five grass genomes with the top hits were aligned in vertical columns (one column for each genome) and were aligned more or less horizontally to syntenic chromosomal regions or linkage groups from other genomes. Maps of each of these chromosome segments with significant hits were prepared using MapChart. The physical distance between marker loci is represented in Mb (mega base pairs) for the sequenced grasses and the distance between marker loci in cM (centiMorgan) for the pearl millet linkage groups in Additional file 9. Lines were drawn between the BLAST hit positions on the chromosomal segments of the five grass genomes and the corresponding marker locus on pearl millet linkage groups to show the syntenic relationships in the figures in Additional file 9, and the results summarized in Table 4.

## Abbreviations

EST: Expressed sequence tag; SSR: Simple sequence repeats; QTL: Quantitative trait locus; DArT: Diversity array technology; SNP: Single nucleotide polymorphism; CISP: Conserved intron spanning primer; RFLP: Restriction fragment length polymorphism; NGS: Next generation sequencing; TUS: Tentative unique sequence; RIL: Recombinant inbred line; RIP: Recombinant inbred population; LG: Linkage group; DAG: Directed acyclic graph; NTR: Normalized transpiration ratio.

## Competing interests

The authors declare that they have no competing interests related to the contents of this manuscript.

## **Authors’ contributions**

CTH was primarily responsible for the coordination of this study. RKV, ORL and MLN have assisted VR in the planning and design of this present study. VR, VV, CTH and RKV were involved in setting up drought stress treatments and isolation of RNA. VR, RKV, TN, SS, TS, AB and ORL were involved in the primer design and bioinformatics work. VR, TN, SS, AS, SK and BRK generated the linkage mapping data and did the linkage mapping. VR, TS and TN constructed the consensus map. VR, CTH, RKV, and ORL were involved in the synteny work. VR, TN, RS, MLN, CTH and ORL wrote the MS and other authors have contributed to it. All the authors have read and approved the final manuscript.

## Supplementary Material

Additional file 1: Table S1 List of putative SNPs identified between ICMB 841-P3 and 863B-P2. The reads from ICMB 841-P3 are prefixed with “X_” and the reads from 863B-P2 are prefixed with “Y_”. The column titled “SNP_variant_in_X” represents the SNP variant in ICMB 841-P3 genotype while the column “SNP_variant_in_Y” represents the SNP variant in 863B-P2 genotype. Click here for file

Additional file 2: Table S2 Forward and reverse primer pair sequences developed from drought-stressed EST data set. Click here for file

Additional file 3: Table S3 Linkage maps for the RIL cross ICMB 841-P3 × 863B-P2 (= RIP A). Click here for file

Additional file 4: Table S4 Linkage maps for the RIL cross H 77/833-2 × PRLT 2/89-33 (= RIP B). Click here for file

Additional file 5: Table S5 Linkage maps for the RIL cross 81B-P6 × ICMP 451-P8 (= RIP C). Click here for file

Additional file 6: Table S6 Linkage maps for the RIL cross PT 732B-P2 × P1449-2-P1 (= RIP D). Click here for file

Additional file 7: Figure S1 Consensus and comparative maps of pearl millet based on four RIL mapping populations. The mapping populations are abbreviated as in the text: RIP A = ICMB 841-P3 × 863B-P2, RIP B = H 77/833-2 × PRLT 2/89-33; RIP C = 81B-P6 × ICMP 451-P8; RIP D = PT 732B-P2 × P1449-2-P1. Click here for file

Additional file 8: Table S7 Linkage group-wise shared markers across the four Recombinant Inbred Populations (RIPs). Click here for file

Additional file 9: Figure S2 Synteny between the pearl millet linkage groups and chromosomes of 5 sequenced grasses. Linkage groups of pearl millet represented as Pg_1 = LG1, Pg_2 = LG2, Pg_3 = LG3, Pg_4 = LG4, Pg_5 = LG5, Pg_6 = LG6, Pg_7 = LG7 and Pg_A = LGA, Chromosomes of foxtail millet named as Si_1 to Si_9, chromosomes of rice as Os_01 to Os_12, chromosomes of sorghum as SBI-01 to SBI-10, chromosomes of maize as Zm_01 to Zm_10, and chromosomes of *Brachypodium* as Bd_1 to Bd_5. BLAST search of the full length EST sequences corresponding to the mapped pearl millet EST-SSR (*Xipes* and *Xicmp*) markers was done separately on each of the 5 sequenced grass genomes. Top hits with *e*-value ≤ E-10 were shown on the chromosomes and lines were drawn between the BLAST hit positions on chromosomes of the 5 grass genomes and corresponding pearl millet linkage groups. The marker names are **bold**, underlined and *italicized* if the pearl millet marker had BLAST hits on 4 or 5 other grass genomes, are **bold** and underlined if the marker had hits on 3 other grass genomes, and are **bold** if the marker had hits on 1 or 2 other grass genomes and are normal font if the marker had no hits on these five grass genomes. “Inverted” in the brackets indicates that the marker order for the respective consensus LG is reversed. Linkage distances (in cM for pearl millet) or physical map positions (in Mb for other grasses) are given on the right side of each bar and the marker names are given on the left side of each bar. Click here for file
