# Supplementary material for: Pearl millet [Pennisetum glaucum (L.) R. Br.] consensus linkage map constructed using four RIL mapping populations and newly developed EST-SSRs
Source: BMC Genomics. 2013 Mar 9;14:159. doi: 10.1186/1471-2164-14-159 (PMC3606598; doi:10.1186/1471-2164-14-159)
Supplement: Additional file 7: Figure S1 — Consensus and comparative maps of pearl millet based on four RIL mapping populations. The mapping populations are abbreviated as in the text: RIP A = ICMB 841-P3 × 863B-P2, RIP B = H 77/833-2 × PRLT 2/89-33; RIP C = 81B-P6 × ICMP 451-P8; RIP D = PT 732B-P2 × P1449-2-P1. [file 1471-2164-14-159-S7.pptx]

## Slide 1
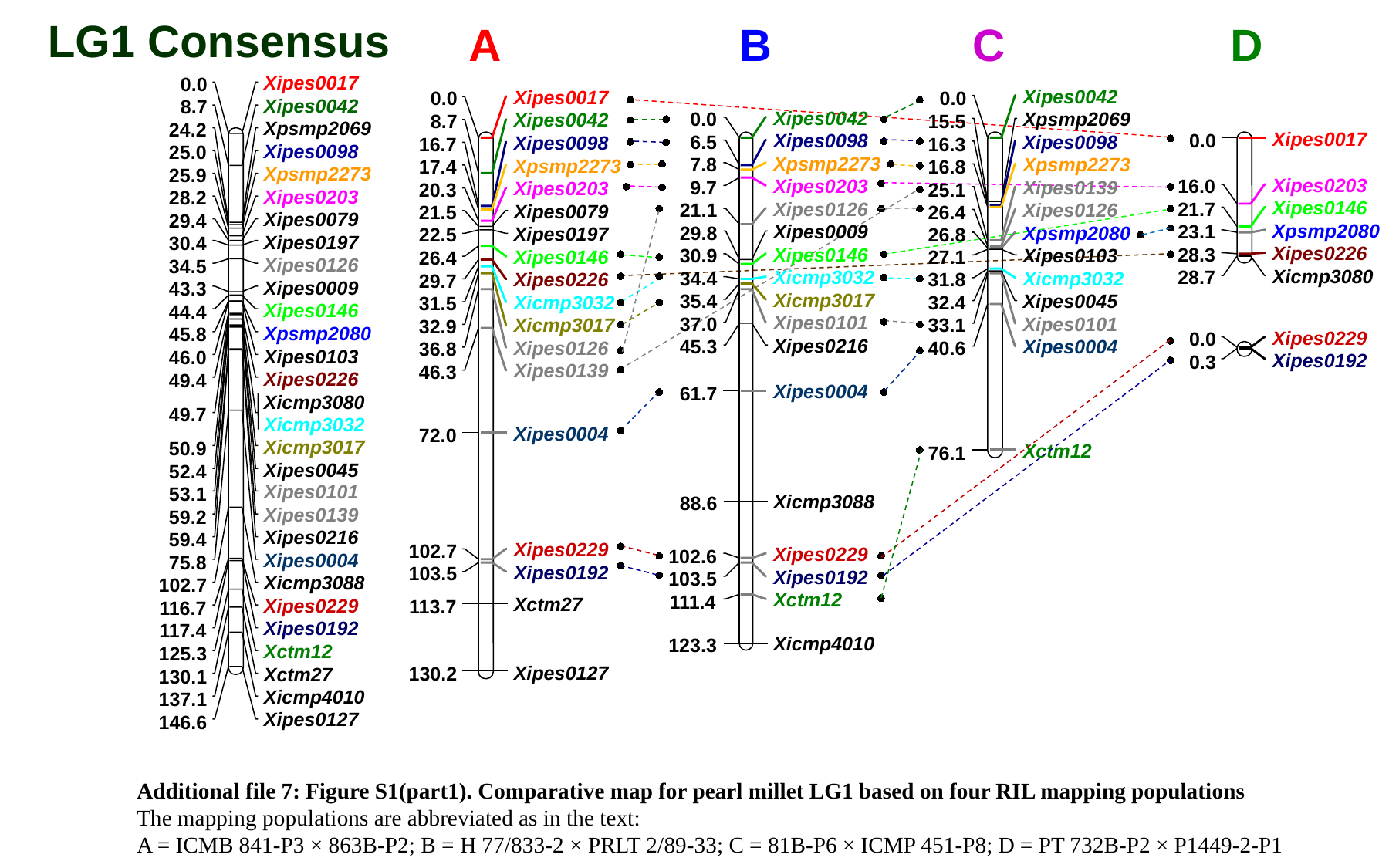

LG1 Consensus
A B C D
Xipes0017
0.0
Xipes0042
8.7
Xpsmp2069
24.2
Xipes0098
25.0
Xpsmp2273
25.9
Xipes0203
28.2
Xipes0079
29.4
Xipes0197
30.4
Xipes0126
34.5
Xipes0009
43.3
Xipes0146
44.4
Xpsmp2080
45.8
Xipes0103
46.0
Xipes0226
49.4
Xicmp3080
49.7
Xicmp3032
Xicmp3017
50.9
Xipes0045
52.4
Xipes0101
53.1
Xipes0139
59.2
Xipes0216
59.4
Xipes0004
75.8
Xicmp3088
102.7
Xipes0229
116.7
Xipes0192
117.4
Xctm12
125.3
Xctm27
130.1
Xicmp4010
137.1
Xipes0127
146.6
Xipes0042
Xipes0017
0.0
0.0
Xipes0042
0.0
Xpsmp2069
Xipes0042
8.7
15.5
Xipes0017
Xipes0098
0.0
6.5
Xipes0098
Xipes0098
16.7
16.3
Xpsmp2273
7.8
Xpsmp2273
Xpsmp2273
17.4
16.8
Xipes0203
Xipes0203
16.0
9.7
Xipes0139
Xipes0203
20.3
25.1
Xipes0146
Xipes0126
21.7
21.1
Xipes0126
Xipes0079
21.5
26.4
Xpsmp2080
Xipes0009
23.1
29.8
Xpsmp2080
Xipes0197
22.5
26.8
Xipes0226
Xipes0146
28.3
30.9
Xipes0103
Xipes0146
27.1
26.4
Xicmp3080
Xicmp3032
28.7
34.4
Xicmp3032
Xipes0226
31.8
29.7
Xicmp3017
35.4
Xipes0045
Xicmp3032
32.4
31.5
Xipes0101
37.0
Xipes0101
Xicmp3017
33.1
32.9
Xipes0229
0.0
Xipes0216
45.3
Xipes0004
Xipes0126
40.6
36.8
Xipes0192
0.3
Xipes0139
46.3
Xipes0004
61.7
Xipes0004
72.0
Xctm12
76.1
Xicmp3088
88.6
Xipes0229
102.7
Xipes0229
102.6
Xipes0192
103.5
Xipes0192
103.5
Xctm12
111.4
Xctm27
113.7
Xicmp4010
123.3
Xipes0127
130.2
Additional file 7: Figure S1(part1). Comparative map for pearl millet LG1 based on four RIL mapping populations
The mapping populations are abbreviated as in the text:
A = ICMB 841-P3 × 863B-P2; B = H 77/833-2 × PRLT 2/89-33; C = 81B-P6 × ICMP 451-P8; D = PT 732B-P2 × P1449-2-P1

## Slide 2
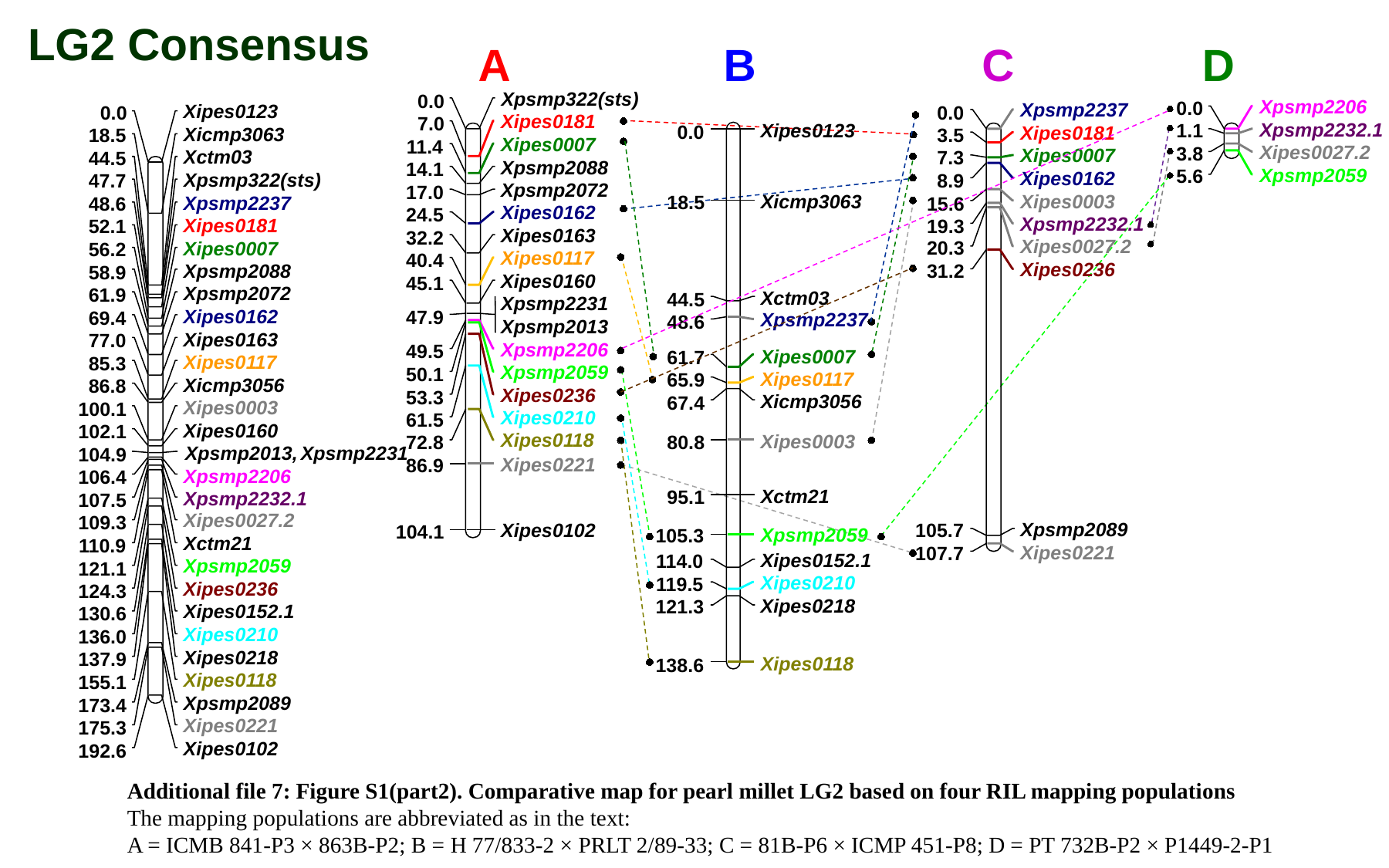

LG2 Consensus
A B C D
Xpsmp322(sts)
0.0
Xpsmp2206
0.0
Xpsmp2237
Xipes0123
0.0
Xicmp3063
18.5
Xctm03
44.5
Xpsmp322(sts)
47.7
Xpsmp2237
48.6
Xipes0181
52.1
Xipes0007
56.2
Xpsmp2088
58.9
Xpsmp2072
61.9
Xipes0162
69.4
Xipes0163
77.0
Xipes0117
85.3
Xicmp3056
86.8
Xipes0003
100.1
Xipes0160
102.1
Xpsmp2013, Xpsmp2231
104.9
Xpsmp2206
106.4
Xpsmp2232.1
107.5
Xipes0027.2
109.3
Xctm21
110.9
Xpsmp2059
121.1
Xipes0236
124.3
Xipes0152.1
130.6
Xipes0210
136.0
Xipes0218
137.9
Xipes0118
155.1
Xpsmp2089
173.4
Xipes0221
175.3
Xipes0102
192.6
0.0
Xipes0181
7.0
Xpsmp2232.1
Xipes0123
1.1
0.0
Xipes0181
3.5
Xipes0007
11.4
Xipes0027.2
3.8
Xipes0007
7.3
Xpsmp2088
14.1
Xpsmp2059
5.6
Xipes0162
8.9
Xpsmp2072
17.0
Xicmp3063
Xipes0003
18.5
15.6
Xipes0162
24.5
Xpsmp2232.1
19.3
Xipes0163
32.2
Xipes0027.2
20.3
Xipes0117
40.4
Xipes0236
31.2
Xipes0160
45.1
Xctm03
44.5
Xpsmp2231
47.9
Xpsmp2237
48.6
Xpsmp2013
Xpsmp2206
49.5
Xipes0007
61.7
Xpsmp2059
50.1
Xipes0117
65.9
Xipes0236
53.3
Xicmp3056
67.4
Xipes0210
61.5
Xipes0118
Xipes0003
72.8
80.8
Xipes0221
86.9
Xctm21
95.1
Xpsmp2089
Xipes0102
105.7
104.1
Xpsmp2059
105.3
Xipes0221
107.7
Xipes0152.1
114.0
Xipes0210
119.5
Xipes0218
121.3
Xipes0118
138.6
Additional file 7: Figure S1(part2). Comparative map for pearl millet LG2 based on four RIL mapping populations
The mapping populations are abbreviated as in the text:
A = ICMB 841-P3 × 863B-P2; B = H 77/833-2 × PRLT 2/89-33; C = 81B-P6 × ICMP 451-P8; D = PT 732B-P2 × P1449-2-P1

## Slide 3
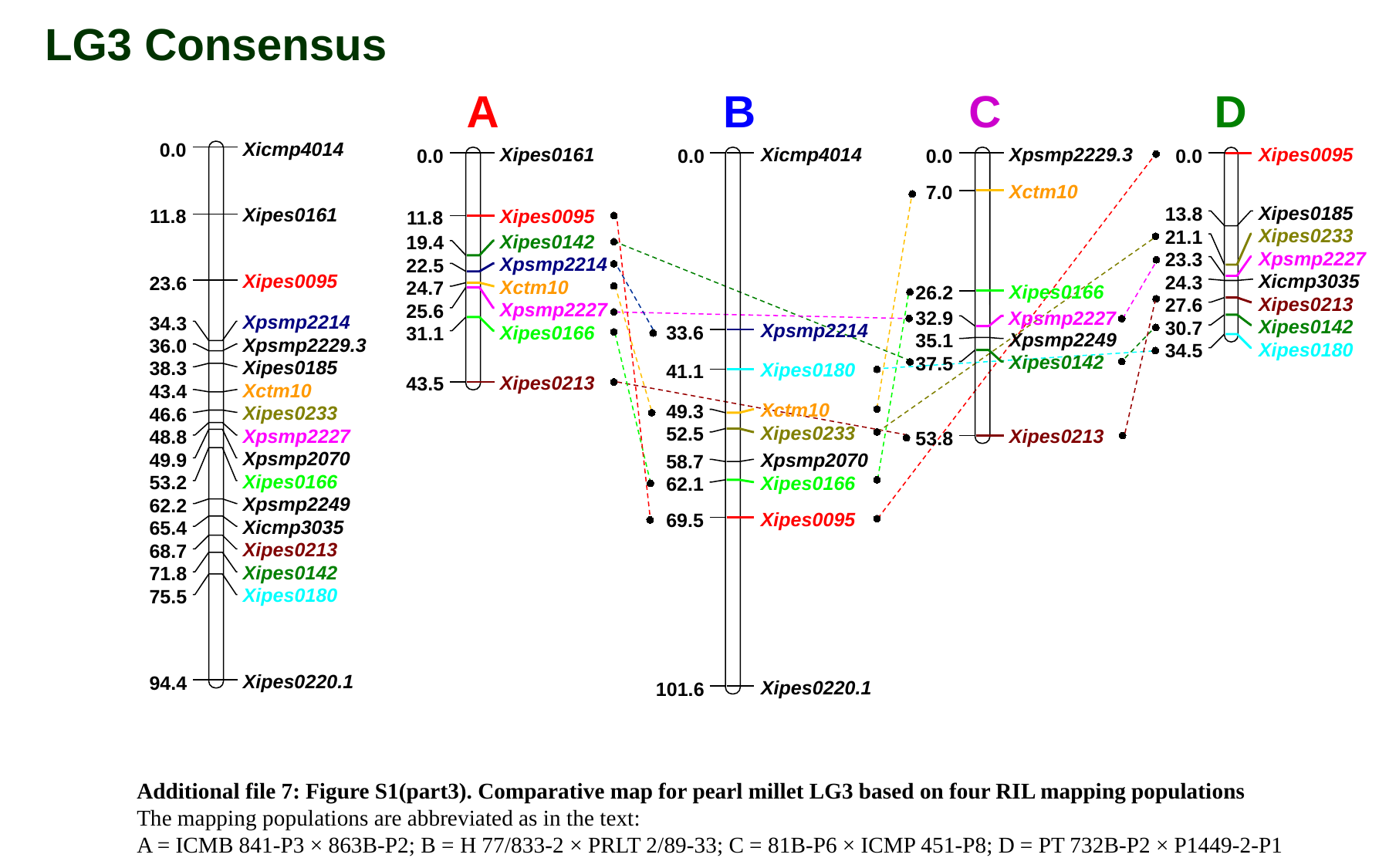

LG3 Consensus
A B C D
Xicmp4014
0.0
Xipes0161
11.8
Xipes0095
23.6
Xpsmp2214
34.3
Xpsmp2229.3
36.0
Xipes0185
38.3
Xctm10
43.4
Xipes0233
46.6
Xpsmp2227
48.8
Xpsmp2070
49.9
Xipes0166
53.2
Xpsmp2249
62.2
Xicmp3035
65.4
Xipes0213
68.7
Xipes0142
71.8
Xipes0180
75.5
Xipes0220.1
94.4
Xipes0161
Xicmp4014
Xpsmp2229.3
Xipes0095
0.0
0.0
0.0
0.0
Xctm10
7.0
Xipes0185
13.8
Xipes0095
11.8
Xipes0233
21.1
Xipes0142
19.4
Xpsmp2227
23.3
Xpsmp2214
22.5
Xicmp3035
24.3
Xctm10
24.7
Xipes0166
26.2
Xipes0213
27.6
Xpsmp2227
25.6
32.9
Xpsmp2227
Xipes0142
30.7
Xpsmp2214
Xipes0166
33.6
31.1
Xpsmp2249
35.1
Xipes0180
34.5
Xipes0142
37.5
Xipes0180
41.1
Xipes0213
43.5
Xctm10
49.3
Xipes0233
52.5
Xipes0213
53.8
Xpsmp2070
58.7
Xipes0166
62.1
Xipes0095
69.5
Xipes0220.1
101.6
Additional file 7: Figure S1(part3). Comparative map for pearl millet LG3 based on four RIL mapping populations
The mapping populations are abbreviated as in the text:
A = ICMB 841-P3 × 863B-P2; B = H 77/833-2 × PRLT 2/89-33; C = 81B-P6 × ICMP 451-P8; D = PT 732B-P2 × P1449-2-P1

## Slide 4
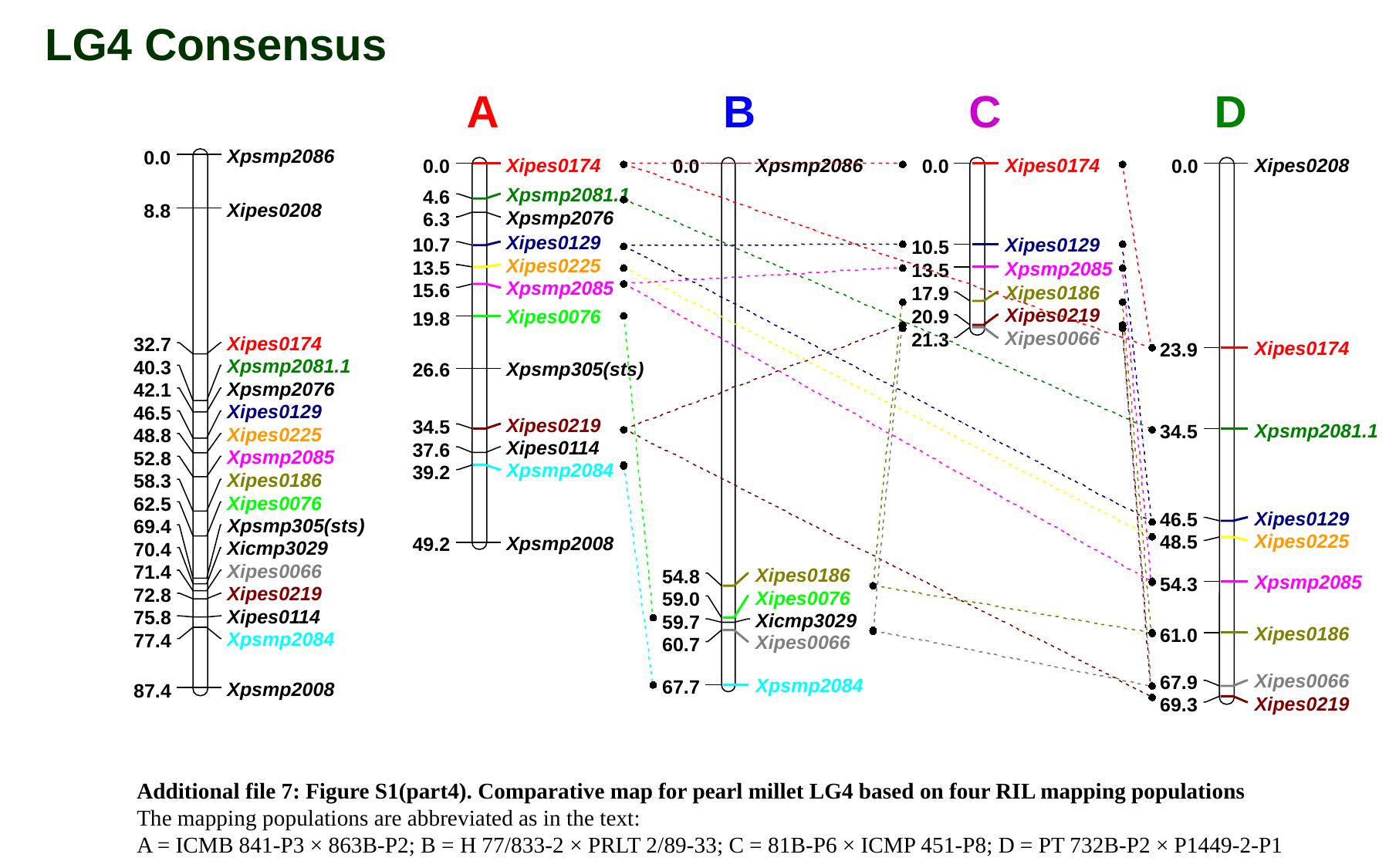

LG4 Consensus
A B C D
Xpsmp2086
0.0
Xipes0208
8.8
Xipes0174
32.7
Xpsmp2081.1
40.3
Xpsmp2076
42.1
Xipes0129
46.5
Xipes0225
48.8
Xpsmp2085
52.8
Xipes0186
58.3
Xipes0076
62.5
Xpsmp305(sts)
69.4
Xicmp3029
70.4
Xipes0066
71.4
Xipes0219
72.8
Xipes0114
75.8
Xpsmp2084
77.4
Xpsmp2008
87.4
Xipes0174
Xpsmp2086
Xipes0174
Xipes0208
0.0
0.0
0.0
0.0
Xpsmp2081.1
4.6
Xpsmp2076
6.3
Xipes0129
10.7
Xipes0129
10.5
Xipes0225
13.5
Xpsmp2085
13.5
Xpsmp2085
15.6
Xipes0186
17.9
Xipes0219
Xipes0076
20.9
19.8
Xipes0066
21.3
Xipes0174
23.9
Xpsmp305(sts)
26.6
Xipes0219
34.5
Xpsmp2081.1
34.5
Xipes0114
37.6
Xpsmp2084
39.2
Xipes0129
46.5
Xipes0225
48.5
Xpsmp2008
49.2
Xipes0186
54.8
Xpsmp2085
54.3
Xipes0076
59.0
Xicmp3029
59.7
Xipes0186
61.0
Xipes0066
60.7
Xipes0066
67.9
Xpsmp2084
67.7
Xipes0219
69.3
Additional file 7: Figure S1(part4). Comparative map for pearl millet LG4 based on four RIL mapping populations
The mapping populations are abbreviated as in the text:
A = ICMB 841-P3 × 863B-P2; B = H 77/833-2 × PRLT 2/89-33; C = 81B-P6 × ICMP 451-P8; D = PT 732B-P2 × P1449-2-P1

## Slide 5
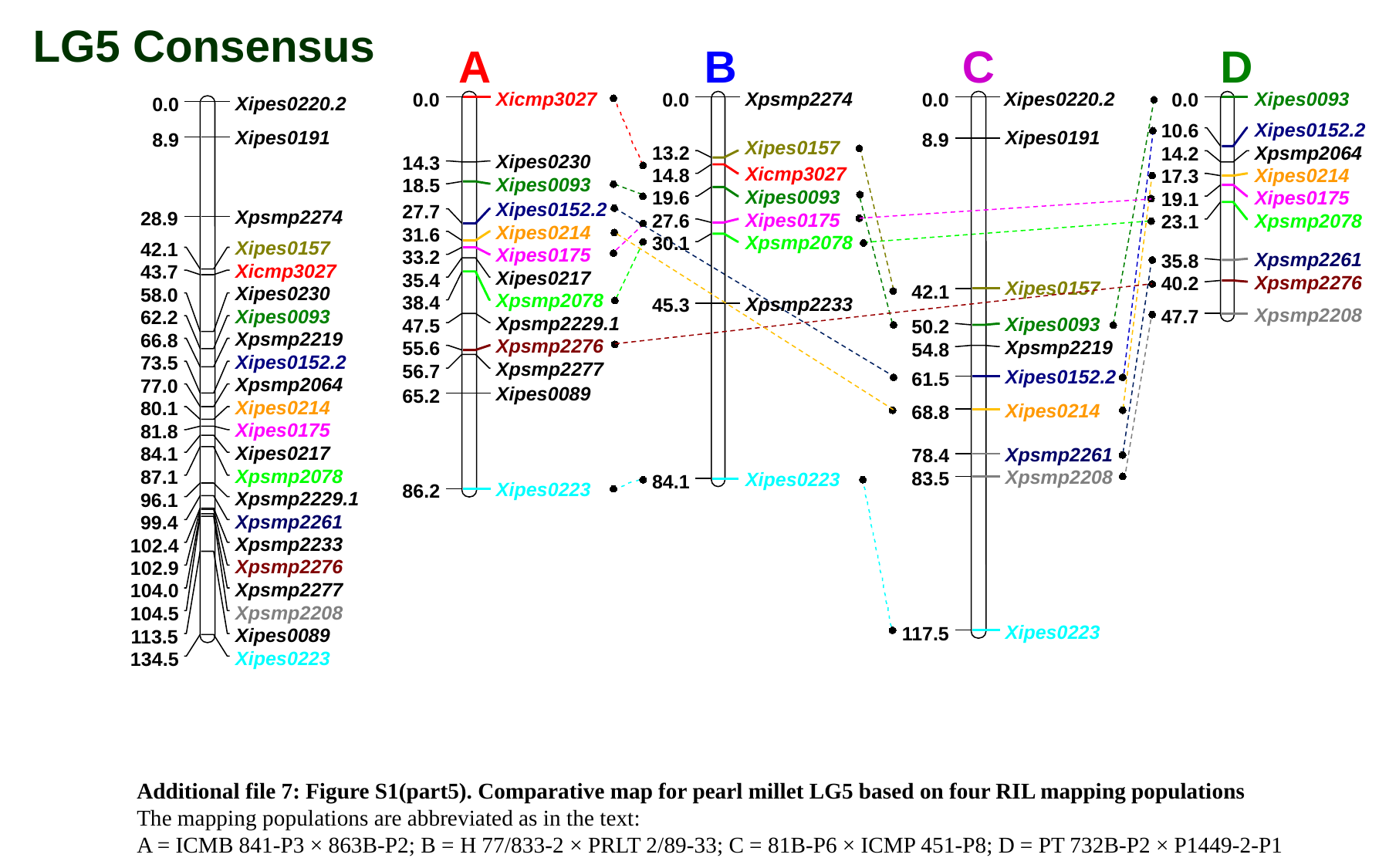

LG5 Consensus
A B C D
Xicmp3027
Xpsmp2274
Xipes0220.2
Xipes0093
0.0
0.0
0.0
0.0
Xipes0220.2
0.0
Xipes0191
8.9
Xpsmp2274
28.9
Xipes0157
42.1
Xicmp3027
43.7
Xipes0230
58.0
Xipes0093
62.2
Xpsmp2219
66.8
Xipes0152.2
73.5
Xpsmp2064
77.0
Xipes0214
80.1
Xipes0175
81.8
Xipes0217
84.1
Xpsmp2078
87.1
Xpsmp2229.1
96.1
Xpsmp2261
99.4
Xpsmp2233
102.4
Xpsmp2276
102.9
Xpsmp2277
104.0
Xpsmp2208
104.5
Xipes0089
113.5
Xipes0223
134.5
Xipes0152.2
10.6
Xipes0191
8.9
Xipes0157
13.2
Xpsmp2064
14.2
Xipes0230
14.3
Xicmp3027
14.8
Xipes0214
17.3
Xipes0093
18.5
Xipes0093
19.6
Xipes0175
19.1
Xipes0152.2
27.7
Xipes0175
27.6
Xpsmp2078
23.1
Xipes0214
31.6
Xpsmp2078
30.1
Xipes0175
33.2
Xpsmp2261
35.8
Xipes0217
35.4
Xpsmp2276
40.2
Xipes0157
42.1
Xpsmp2078
38.4
Xpsmp2233
45.3
Xpsmp2208
47.7
Xpsmp2229.1
Xipes0093
47.5
50.2
Xpsmp2276
Xpsmp2219
55.6
54.8
Xpsmp2277
56.7
Xipes0152.2
61.5
Xipes0089
65.2
Xipes0214
68.8
Xpsmp2261
78.4
Xpsmp2208
83.5
Xipes0223
84.1
Xipes0223
86.2
Xipes0223
117.5
Additional file 7: Figure S1(part5). Comparative map for pearl millet LG5 based on four RIL mapping populations
The mapping populations are abbreviated as in the text:
A = ICMB 841-P3 × 863B-P2; B = H 77/833-2 × PRLT 2/89-33; C = 81B-P6 × ICMP 451-P8; D = PT 732B-P2 × P1449-2-P1

## Slide 6
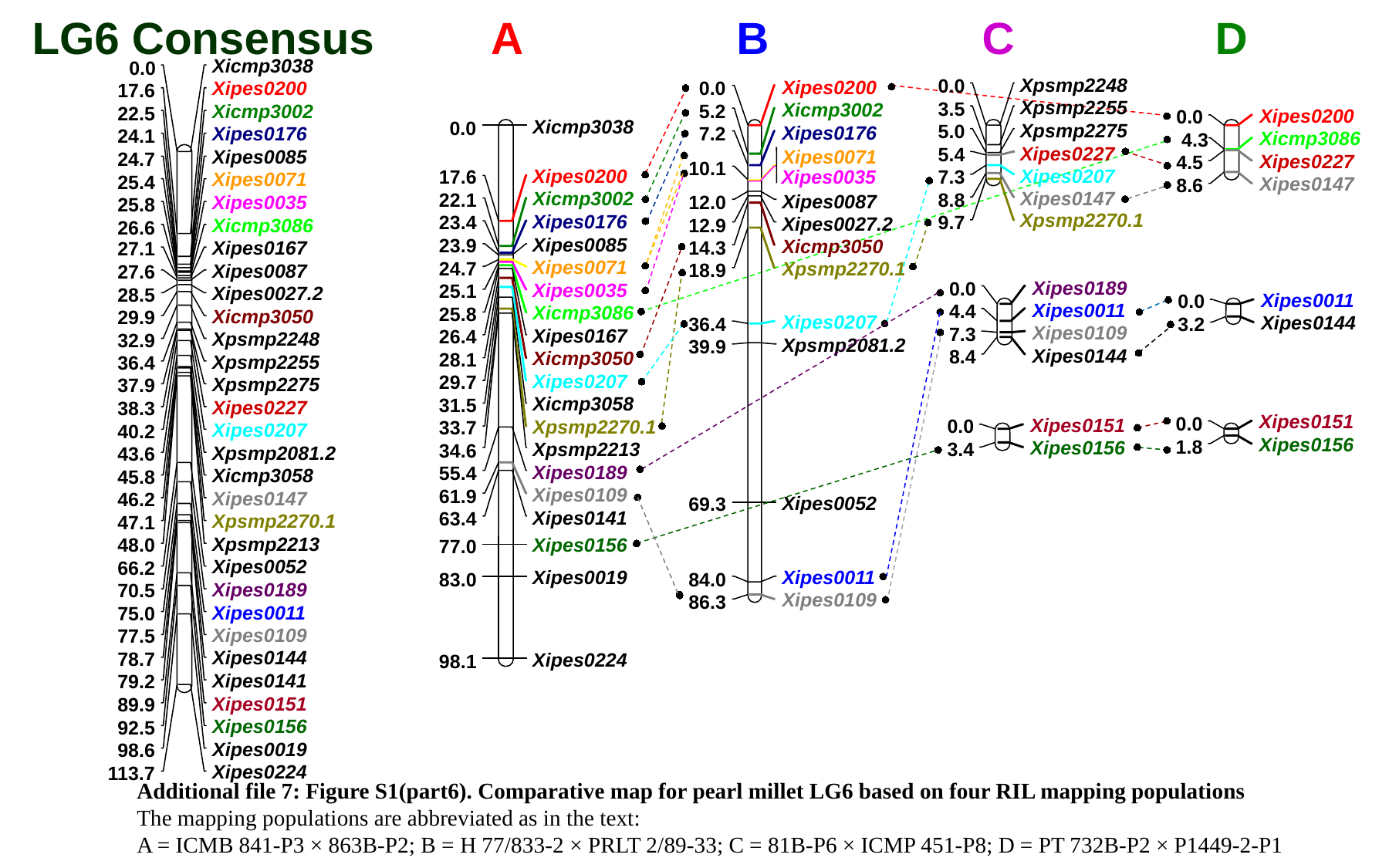

LG6 Consensus
A B C D
Xicmp3038
0.0
Xipes0200
17.6
Xicmp3002
22.5
Xipes0176
24.1
Xipes0085
24.7
Xipes0071
25.4
Xipes0035
25.8
Xicmp3086
26.6
Xipes0167
27.1
Xipes0087
27.6
Xipes0027.2
28.5
Xicmp3050
29.9
Xpsmp2248
32.9
Xpsmp2255
36.4
Xpsmp2275
37.9
Xipes0227
38.3
Xipes0207
40.2
Xpsmp2081.2
43.6
Xicmp3058
45.8
Xipes0147
46.2
Xpsmp2270.1
47.1
Xpsmp2213
48.0
Xipes0052
66.2
Xipes0189
70.5
Xipes0011
75.0
Xipes0109
77.5
Xipes0144
78.7
Xipes0141
79.2
Xipes0151
89.9
Xipes0156
92.5
Xipes0019
98.6
Xipes0224
113.7
Xpsmp2248
0.0
Xipes0200
0.0
Xpsmp2255
3.5
Xicmp3002
5.2
Xipes0200
0.0
Xicmp3038
0.0
Xpsmp2275
5.0
Xipes0176
7.2
Xicmp3086
 4.3
Xipes0227
5.4
Xipes0071
Xipes0227
4.5
10.1
Xipes0200
Xipes0207
Xipes0035
17.6
7.3
Xipes0147
8.6
Xicmp3002
Xipes0147
22.1
8.8
Xipes0087
12.0
Xpsmp2270.1
Xipes0176
23.4
9.7
Xipes0027.2
12.9
Xipes0085
23.9
Xicmp3050
14.3
Xipes0071
24.7
Xpsmp2270.1
18.9
Xipes0189
0.0
Xipes0035
25.1
Xipes0011
0.0
Xipes0011
4.4
Xicmp3086
25.8
Xipes0207
Xipes0144
3.2
36.4
Xipes0109
7.3
Xipes0167
26.4
Xpsmp2081.2
39.9
Xipes0144
8.4
Xicmp3050
28.1
Xipes0207
29.7
Xicmp3058
31.5
Xipes0151
0.0
Xipes0151
0.0
Xpsmp2270.1
33.7
Xipes0156
1.8
Xipes0156
3.4
Xpsmp2213
34.6
Xipes0189
55.4
Xipes0109
61.9
Xipes0052
69.3
Xipes0141
63.4
Xipes0156
77.0
Xipes0019
Xipes0011
83.0
84.0
Xipes0109
86.3
Xipes0224
98.1
Additional file 7: Figure S1(part6). Comparative map for pearl millet LG6 based on four RIL mapping populations
The mapping populations are abbreviated as in the text:
A = ICMB 841-P3 × 863B-P2; B = H 77/833-2 × PRLT 2/89-33; C = 81B-P6 × ICMP 451-P8; D = PT 732B-P2 × P1449-2-P1

## Slide 7
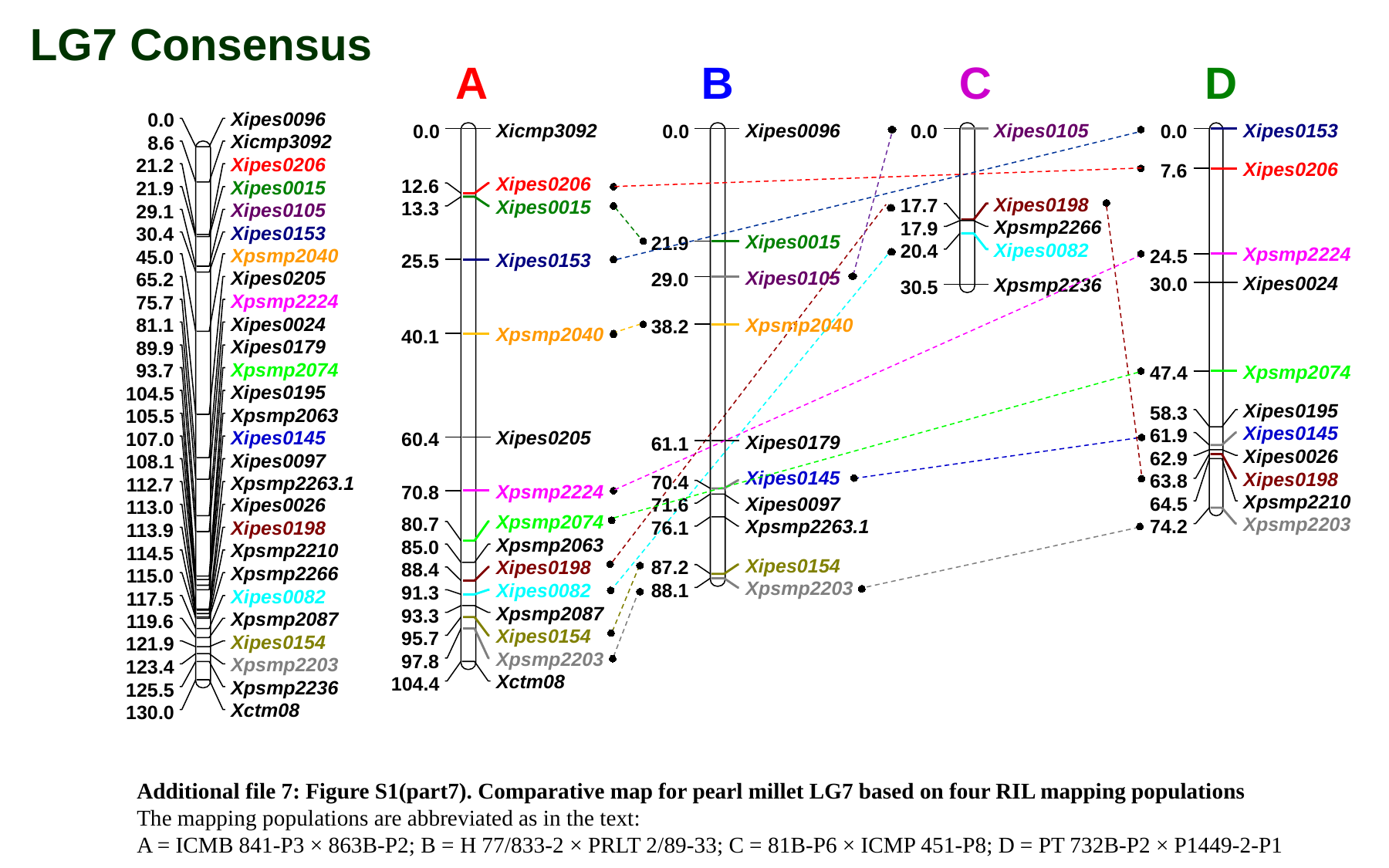

LG7 Consensus
A B C D
Xipes0096
0.0
Xicmp3092
8.6
Xipes0206
21.2
Xipes0015
21.9
Xipes0105
29.1
Xipes0153
30.4
Xpsmp2040
45.0
Xipes0205
65.2
Xpsmp2224
75.7
Xipes0024
81.1
Xipes0179
89.9
Xpsmp2074
93.7
Xipes0195
104.5
Xpsmp2063
105.5
Xipes0145
107.0
Xipes0097
108.1
Xpsmp2263.1
112.7
Xipes0026
113.0
Xipes0198
113.9
Xpsmp2210
114.5
Xpsmp2266
115.0
Xipes0082
117.5
Xpsmp2087
119.6
Xipes0154
121.9
Xpsmp2203
123.4
Xpsmp2236
125.5
Xctm08
130.0
Xicmp3092
Xipes0096
Xipes0105
Xipes0153
0.0
0.0
0.0
0.0
Xipes0206
7.6
Xipes0206
12.6
Xipes0198
17.7
Xipes0015
13.3
Xpsmp2266
17.9
Xipes0015
21.9
Xipes0082
20.4
Xpsmp2224
24.5
Xipes0153
25.5
Xipes0105
29.0
Xipes0024
30.0
Xpsmp2236
30.5
Xpsmp2040
38.2
Xpsmp2040
40.1
Xpsmp2074
47.4
Xipes0195
58.3
Xipes0145
61.9
Xipes0205
60.4
Xipes0179
61.1
Xipes0026
62.9
Xipes0145
Xipes0198
63.8
70.4
Xpsmp2224
70.8
Xpsmp2210
Xipes0097
64.5
71.6
Xpsmp2074
80.7
Xpsmp2203
Xpsmp2263.1
74.2
76.1
Xpsmp2063
85.0
Xipes0154
Xipes0198
87.2
88.4
Xpsmp2203
Xipes0082
88.1
91.3
Xpsmp2087
93.3
Xipes0154
95.7
Xpsmp2203
97.8
Xctm08
104.4
Additional file 7: Figure S1(part7). Comparative map for pearl millet LG7 based on four RIL mapping populations
The mapping populations are abbreviated as in the text:
A = ICMB 841-P3 × 863B-P2; B = H 77/833-2 × PRLT 2/89-33; C = 81B-P6 × ICMP 451-P8; D = PT 732B-P2 × P1449-2-P1
