# Supplementary material for: Pearl millet [Pennisetum glaucum (L.) R. Br.] consensus linkage map constructed using four RIL mapping populations and newly developed EST-SSRs
Source: BMC Genomics. 2013 Mar 9;14:159. doi: 10.1186/1471-2164-14-159 (PMC3606598; doi:10.1186/1471-2164-14-159)
Supplement: Additional file 9: Figure S2 — Synteny between the pearl millet linkage groups and chromosomes of 5 sequenced grasses. Linkage groups of pearl millet represented as Pg_1 = LG1, Pg_2 = LG2, Pg_3 = LG3, Pg_4 = LG4, Pg_5 = LG5, Pg_6 = LG6, Pg_7 = LG7 and Pg_A = LGA, Chromosomes of foxtail millet named as Si_1 to Si_9, chromosomes of rice as Os_01 to Os_12, chromosomes of sorghum as SBI-01 to SBI-10, chromosomes of maize as Zm_01 to Zm_10, and chromosomes of Brachypodium as Bd_1 to Bd_5. BLAST search of the full length EST sequences corresponding to the mapped pearl millet EST-SSR (Xipes and Xicmp) markers was done separately on each of the 5 sequenced grass genomes. Top hits with e-value ≤ E-10 were shown on the chromosomes and lines were drawn between the BLAST hit positions on chromosomes of the 5 grass genomes and corresponding pearl millet linkage groups. The marker names are bold, underlined and italicized if the pearl millet marker had BLAST hits on 4 or 5 other grass genomes, are bold and underlined if the marker had hits on 3 other grass genomes, and are bold if the marker had hits on 1 or 2 other grass genomes and are normal font if the marker had no hits on these five grass genomes. “Inverted” in the brackets indicates that the marker order for the respective consensus LG is reversed. Linkage distances (in cM for pearl millet) or physical map positions (in Mb for other grasses) are given on the right side of each bar and the marker names are given on the left side of each bar. [file 1471-2164-14-159-S9.pptx]

## Slide 1
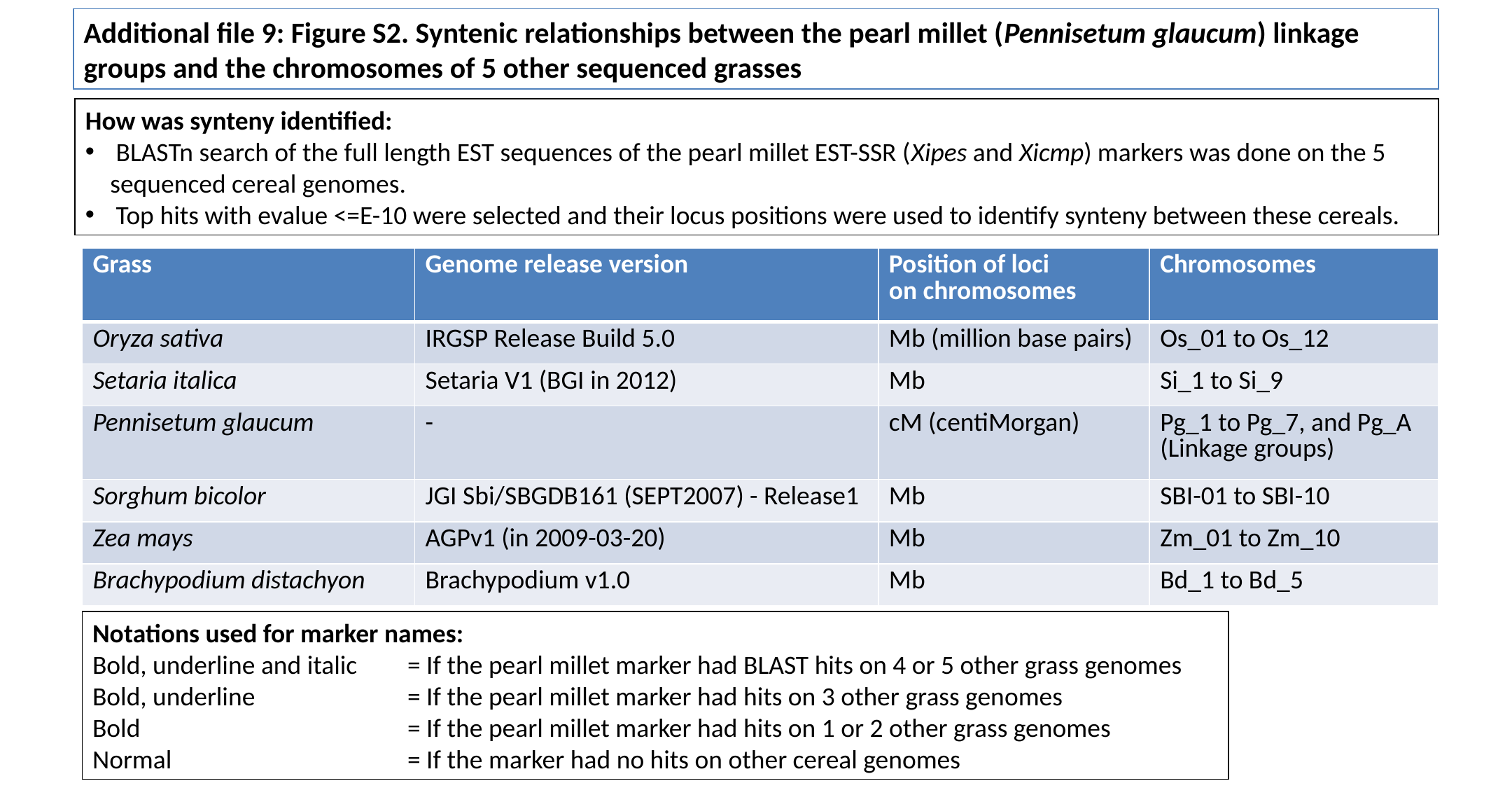

Additional file 9: Figure S2. Syntenic relationships between the pearl millet (Pennisetum glaucum) linkage groups and the chromosomes of 5 other sequenced grasses
How was synteny identified:
 BLASTn search of the full length EST sequences of the pearl millet EST-SSR (Xipes and Xicmp) markers was done on the 5 sequenced cereal genomes.
 Top hits with evalue <=E-10 were selected and their locus positions were used to identify synteny between these cereals.
| Grass | Genome release version | Position of loci on chromosomes | Chromosomes |
| --- | --- | --- | --- |
| Oryza sativa | IRGSP Release Build 5.0 | Mb (million base pairs) | Os\_01 to Os\_12 |
| Setaria italica | Setaria V1 (BGI in 2012) | Mb | Si\_1 to Si\_9 |
| Pennisetum glaucum | - | cM (centiMorgan) | Pg\_1 to Pg\_7, and Pg\_A (Linkage groups) |
| Sorghum bicolor | JGI Sbi/SBGDB161 (SEPT2007) - Release1 | Mb | SBI-01 to SBI-10 |
| Zea mays | AGPv1 (in 2009-03-20) | Mb | Zm\_01 to Zm\_10 |
| Brachypodium distachyon | Brachypodium v1.0 | Mb | Bd\_1 to Bd\_5 |
Notations used for marker names:
Bold, underline and italic 	= If the pearl millet marker had BLAST hits on 4 or 5 other grass genomes
Bold, underline		= If the pearl millet marker had hits on 3 other grass genomes
Bold			= If the pearl millet marker had hits on 1 or 2 other grass genomes
Normal 			= If the marker had no hits on other cereal genomes

## Slide 2
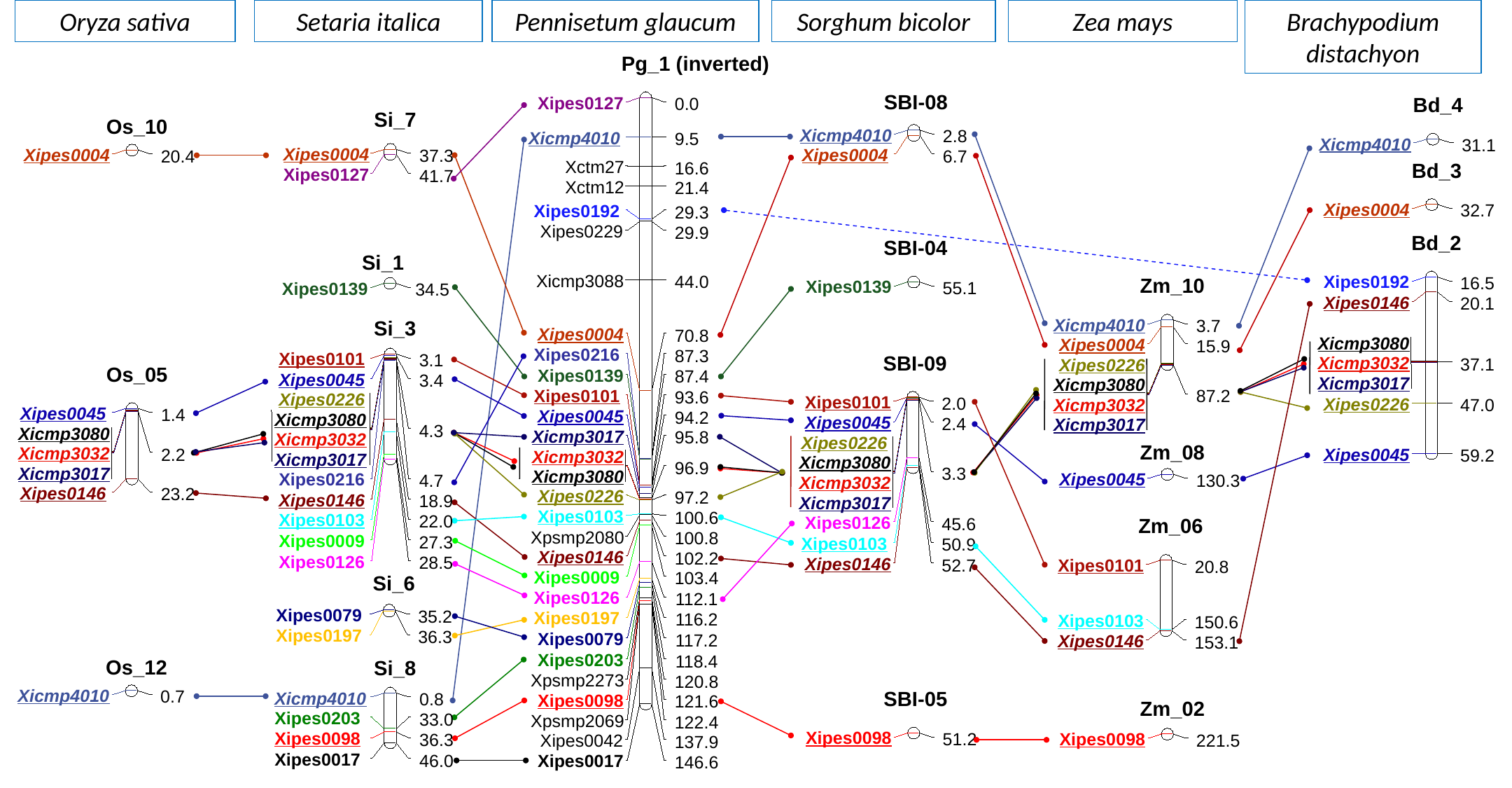

Oryza sativa
Setaria italica
Pennisetum glaucum
Sorghum bicolor
Zea mays
Brachypodium distachyon
Pg_1 (inverted)
Xipes0127
0.0
Xicmp4010
9.5
Xctm27
16.6
Xctm12
21.4
Xipes0192
29.3
Xipes0229
29.9
Xicmp3088
44.0
Xipes0004
70.8
Xipes0216
87.3
Xipes0139
87.4
Xipes0101
93.6
Xipes0045
94.2
Xicmp3017
95.8
Xicmp3032
96.9
Xicmp3080
Xipes0226
97.2
Xipes0103
100.6
Xpsmp2080
100.8
Xipes0146
102.2
Xipes0009
103.4
Xipes0126
112.1
Xipes0197
116.2
Xipes0079
117.2
Xipes0203
118.4
Xpsmp2273
120.8
Xipes0098
121.6
Xpsmp2069
122.4
Xipes0042
137.9
Xipes0017
146.6
SBI-08
Bd_4
Xicmp4010
31.1
Si_7
Os_10
Xipes0004
20.4
Xicmp4010
2.8
Xipes0004
6.7
Xipes0004
37.3
Xipes0127
41.7
Bd_3
Xipes0004
32.7
Bd_2
Xipes0192
16.5
Xipes0146
20.1
Xicmp3080
Xicmp3032
37.1
Xicmp3017
Xipes0226
47.0
Xipes0045
59.2
SBI-04
Xipes0139
55.1
Si_1
Xipes0139
34.5
Zm_10
Xicmp4010
3.7
Xipes0004
15.9
Xipes0226
Xicmp3080
87.2
Xicmp3032
Xicmp3017
Si_3
Xipes0101
3.1
Xipes0045
3.4
Xipes0226
Xicmp3080
4.3
Xicmp3032
Xicmp3017
Xipes0216
4.7
Xipes0146
18.9
Xipes0103
22.0
Xipes0009
27.3
Xipes0126
28.5
SBI-09
Xipes0101
2.0
Xipes0045
2.4
Xipes0226
Xicmp3080
3.3
Xicmp3032
Xicmp3017
Xipes0126
45.6
Xipes0103
50.9
Xipes0146
52.7
Os_05
Xipes0045
1.4
Xicmp3080
Xicmp3032
2.2
Xicmp3017
Xipes0146
23.2
Zm_08
Xipes0045
130.3
Zm_06
Xipes0101
20.8
Xipes0103
150.6
Xipes0146
153.1
Si_6
Xipes0079
35.2
Xipes0197
36.3
Os_12
Xicmp4010
0.7
Si_8
SBI-05
Xipes0098
51.2
Xicmp4010
0.8
Xipes0203
33.0
Xipes0098
36.3
Xipes0017
46.0
Zm_02
Xipes0098
221.5

## Slide 3
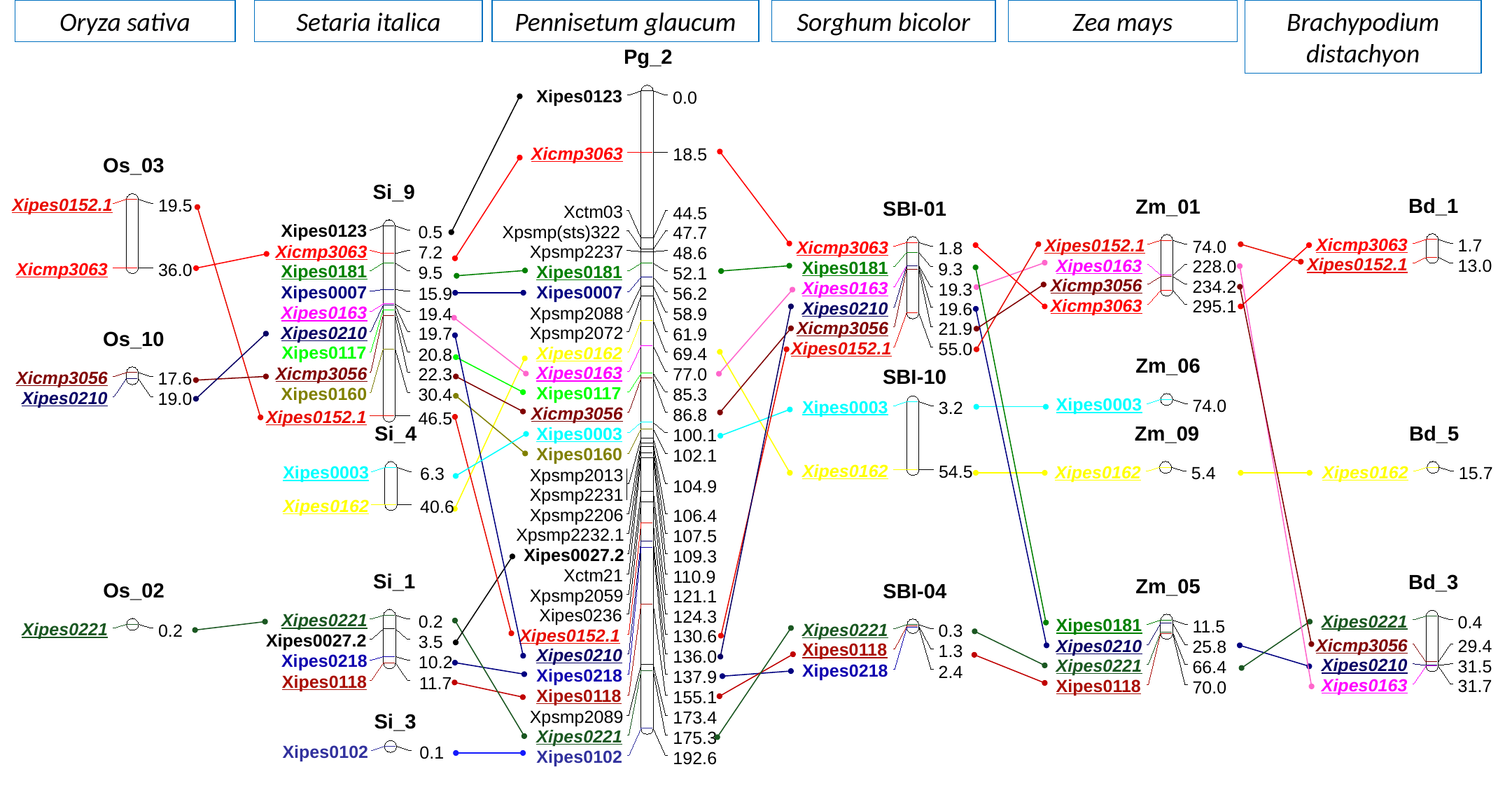

Oryza sativa
Setaria italica
Pennisetum glaucum
Sorghum bicolor
Zea mays
Brachypodium distachyon
Pg_2
Xipes0123
0.0
Xicmp3063
18.5
Xctm03
44.5
Xpsmp(sts)322
47.7
Xpsmp2237
48.6
Xipes0181
52.1
Xipes0007
56.2
Xpsmp2088
58.9
Xpsmp2072
61.9
Xipes0162
69.4
Xipes0163
77.0
Xipes0117
85.3
Xicmp3056
86.8
Xipes0003
100.1
Xipes0160
102.1
Xpsmp2013
104.9
Xpsmp2231
Xpsmp2206
106.4
Xpsmp2232.1
107.5
Xipes0027.2
109.3
Xctm21
110.9
Xpsmp2059
121.1
Xipes0236
124.3
Xipes0152.1
130.6
Xipes0210
136.0
Xipes0218
137.9
Xipes0118
155.1
Xpsmp2089
173.4
Xipes0221
175.3
Xipes0102
192.6
Os_03
Xipes0152.1
19.5
Xicmp3063
36.0
Si_9
Bd_1
Xicmp3063
1.7
Xipes0152.1
13.0
Zm_01
Xipes0152.1
74.0
Xipes0163
228.0
Xicmp3056
234.2
Xicmp3063
295.1
SBI-01
Xicmp3063
1.8
Xipes0181
9.3
Xipes0163
19.3
Xipes0210
19.6
Xicmp3056
21.9
Xipes0152.1
55.0
Xipes0123
0.5
Xicmp3063
7.2
Xipes0181
9.5
Xipes0007
15.9
Xipes0163
19.4
Xipes0210
19.7
Xipes0117
20.8
Xicmp3056
22.3
Xipes0160
30.4
Xipes0152.1
46.5
Os_10
Xicmp3056
17.6
Xipes0210
19.0
Zm_06
Xipes0003
74.0
SBI-10
Xipes0003
3.2
Xipes0162
54.5
Zm_09
Xipes0162
5.4
Bd_5
Xipes0162
15.7
Si_4
Xipes0003
6.3
Xipes0162
40.6
Si_1
Bd_3
Xipes0221
0.4
Xicmp3056
29.4
Xipes0210
31.5
Xipes0163
31.7
Zm_05
Xipes0181
11.5
Xipes0210
25.8
Xipes0221
66.4
Xipes0118
70.0
Os_02
Xipes0221
0.2
SBI-04
Xipes0221
0.3
Xipes0118
1.3
Xipes0218
2.4
Xipes0221
0.2
Xipes0027.2
3.5
Xipes0218
10.2
Xipes0118
11.7
Si_3
Xipes0102
0.1

## Slide 4
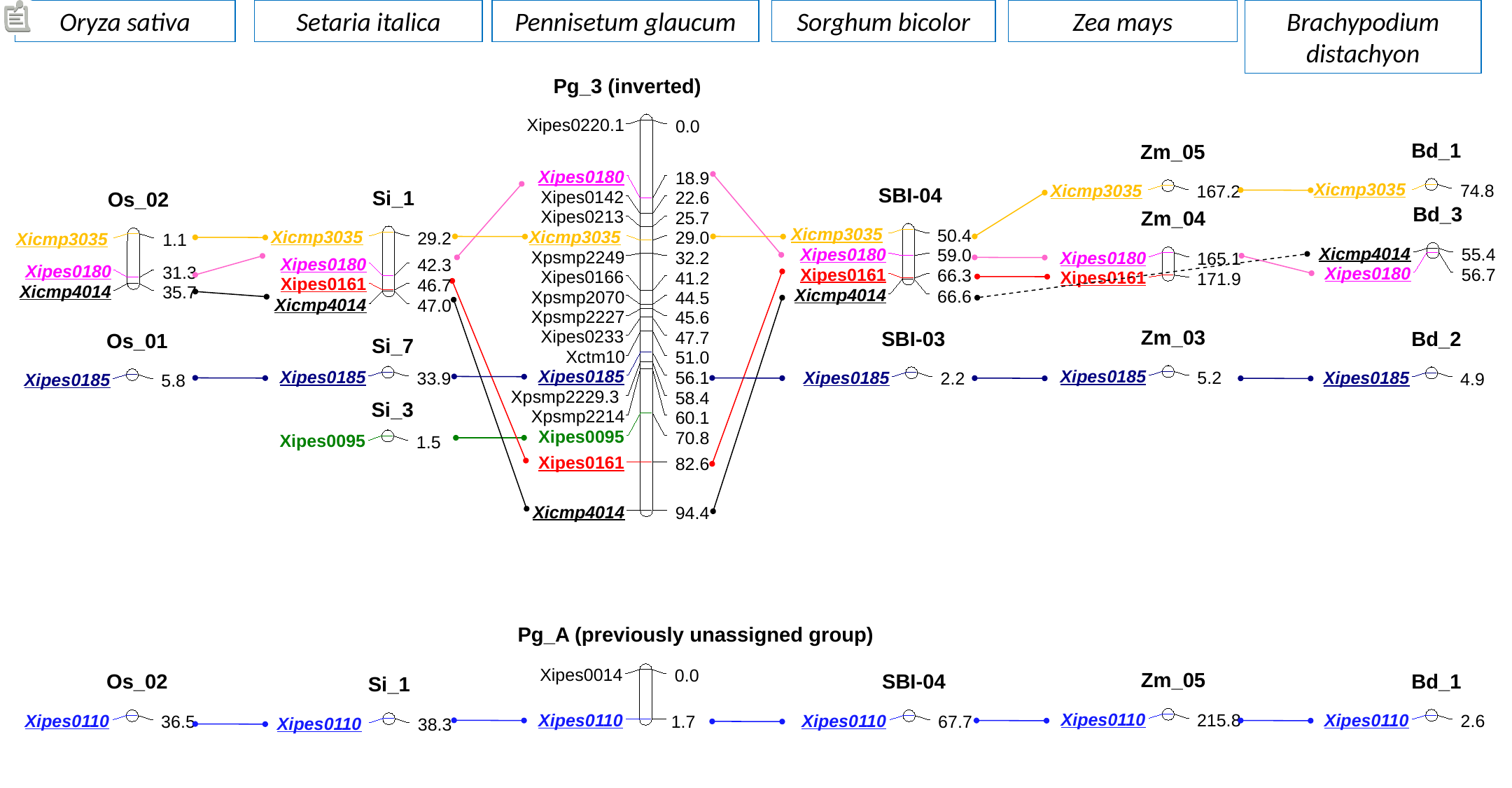

Oryza sativa
Setaria italica
Pennisetum glaucum
Sorghum bicolor
Zea mays
Brachypodium distachyon
Pg_3 (inverted)
Xipes0220.1
0.0
Xipes0180
18.9
Xipes0142
22.6
Xipes0213
25.7
Xicmp3035
29.0
Xpsmp2249
32.2
Xipes0166
41.2
Xpsmp2070
44.5
Xpsmp2227
45.6
Xipes0233
47.7
Xctm10
51.0
Xipes0185
56.1
Xpsmp2229.3
58.4
Xpsmp2214
60.1
Xipes0095
70.8
Xipes0161
82.6
Xicmp4014
94.4
Bd_1
Xicmp3035
74.8
Zm_05
Xicmp3035
167.2
SBI-04
Xicmp3035
50.4
Xipes0180
59.0
Xipes0161
66.3
Xicmp4014
66.6
Si_1
Os_02
Xicmp3035
1.1
Xipes0180
31.3
Xicmp4014
35.7
Bd_3
Xicmp4014
55.4
Xipes0180
56.7
Zm_04
Xipes0180
165.1
Xipes0161
171.9
Xicmp3035
29.2
Xipes0180
42.3
Xipes0161
46.7
Xicmp4014
47.0
Zm_03
Xipes0185
5.2
SBI-03
Xipes0185
2.2
Bd_2
Xipes0185
4.9
Os_01
Xipes0185
5.8
Si_7
Xipes0185
33.9
Si_3
Xipes0095
1.5
Pg_A (previously unassigned group)
Xipes0014
0.0
Xipes0110
1.7
Zm_05
Xipes0110
215.8
Bd_1
Xipes0110
2.6
Os_02
Xipes0110
36.5
SBI-04
Xipes0110
67.7
Si_1
Xipes0110
38.3

## Slide 5
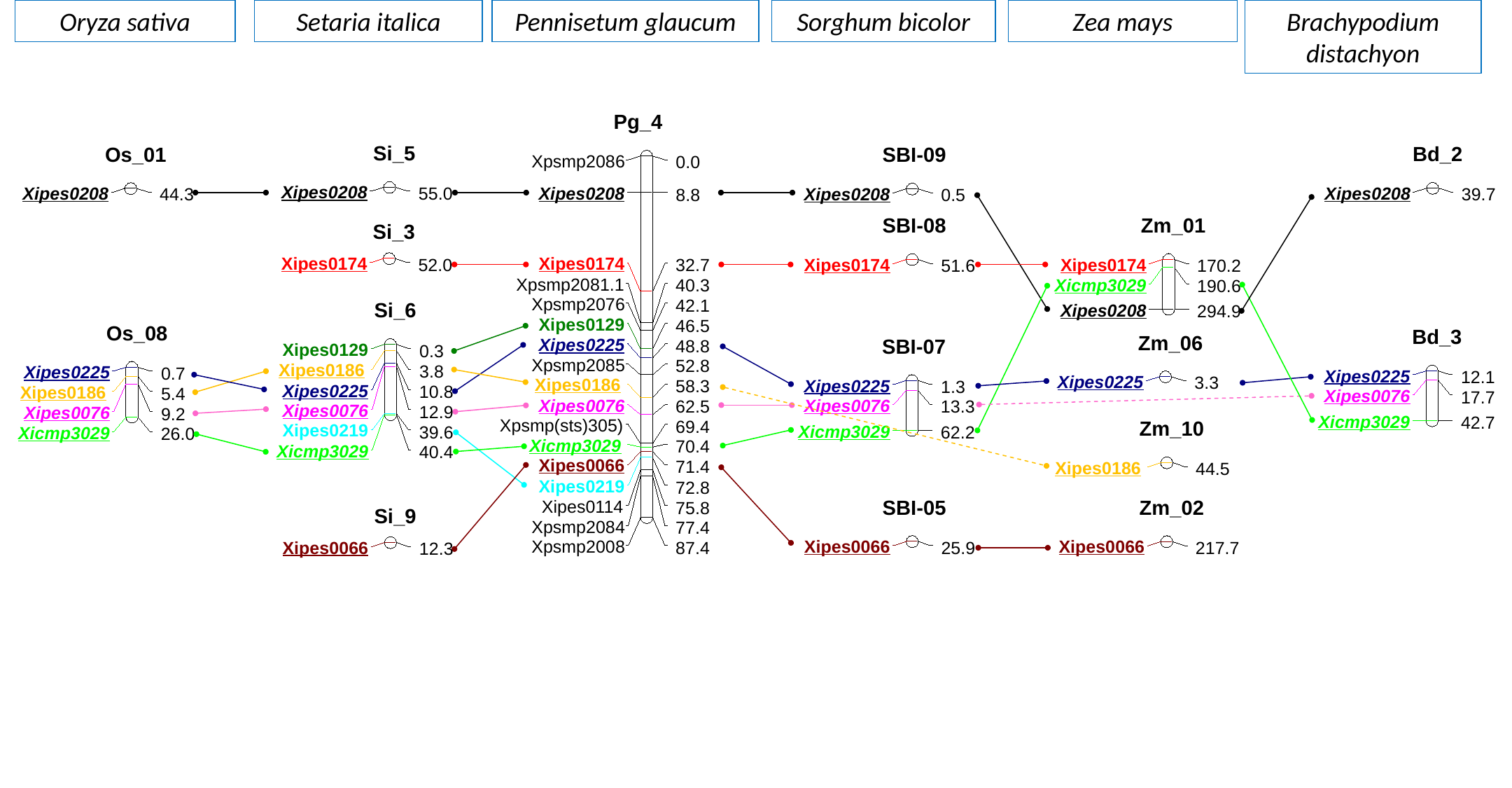

Oryza sativa
Setaria italica
Pennisetum glaucum
Sorghum bicolor
Zea mays
Brachypodium distachyon
Pg_4
Xpsmp2086
0.0
Xipes0208
8.8
Xipes0174
32.7
Xpsmp2081.1
40.3
Xpsmp2076
42.1
Xipes0129
46.5
Xipes0225
48.8
Xpsmp2085
52.8
Xipes0186
58.3
Xipes0076
62.5
Xpsmp(sts)305)
69.4
Xicmp3029
70.4
Xipes0066
71.4
Xipes0219
72.8
Xipes0114
75.8
Xpsmp2084
77.4
Xpsmp2008
87.4
Si_5
Xipes0208
55.0
Bd_2
Xipes0208
39.7
Os_01
Xipes0208
44.3
SBI-09
Xipes0208
0.5
SBI-08
Xipes0174
51.6
Zm_01
Xipes0174
170.2
Xicmp3029
190.6
Xipes0208
294.9
Si_3
Xipes0174
52.0
Si_6
Xipes0129
0.3
Xipes0186
3.8
Xipes0225
10.8
Xipes0076
12.9
Xipes0219
39.6
Xicmp3029
40.4
Os_08
Xipes0225
0.7
Xipes0186
5.4
Xipes0076
9.2
Xicmp3029
26.0
Bd_3
Xipes0225
12.1
Xipes0076
17.7
Xicmp3029
42.7
Zm_06
Xipes0225
3.3
SBI-07
Xipes0225
1.3
Xipes0076
13.3
Xicmp3029
62.2
Zm_10
Xipes0186
44.5
SBI-05
Xipes0066
25.9
Zm_02
Xipes0066
217.7
Si_9
Xipes0066
12.3

## Slide 6
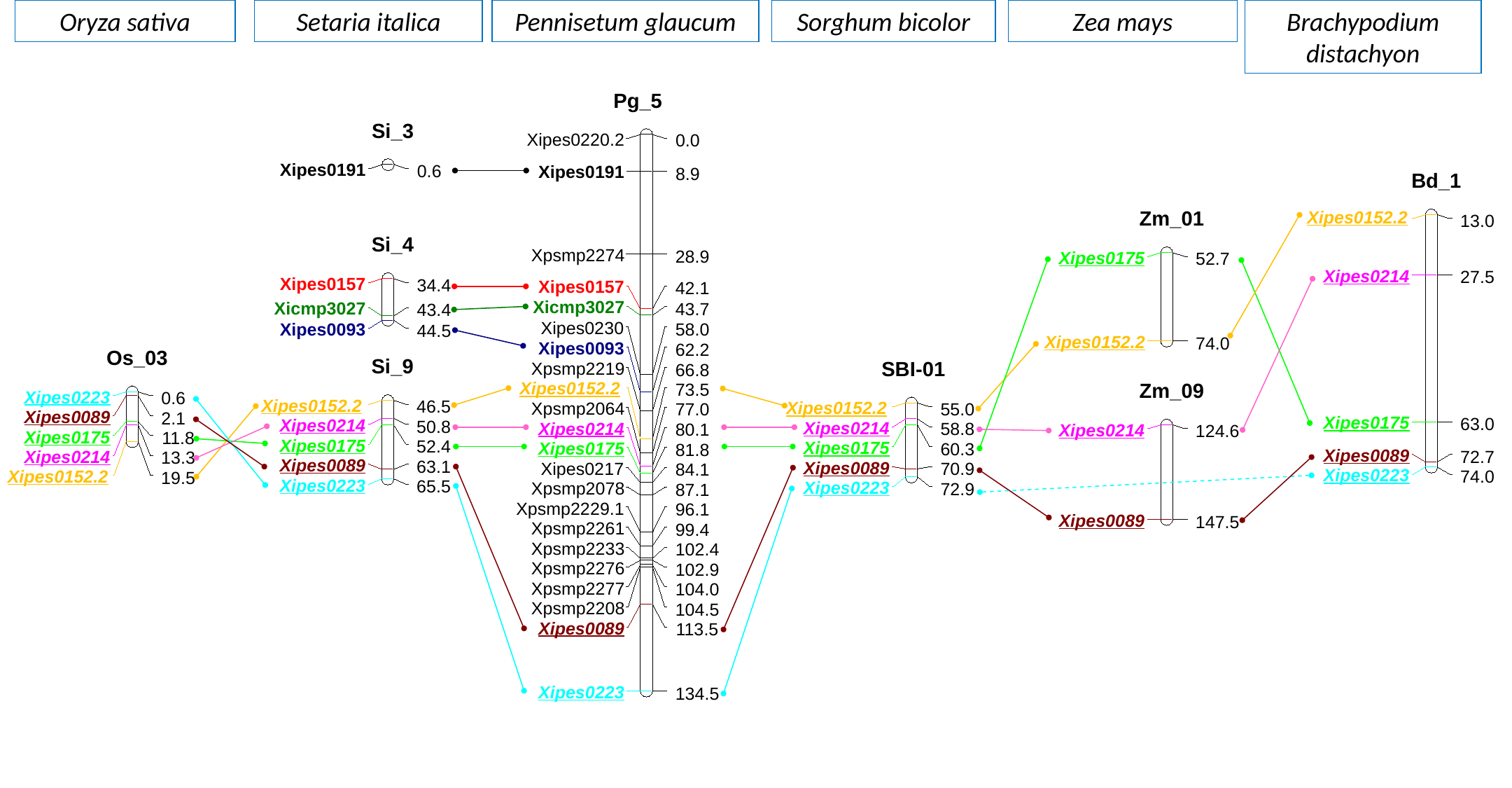

Oryza sativa
Setaria italica
Pennisetum glaucum
Sorghum bicolor
Zea mays
Brachypodium distachyon
Pg_5
Xipes0220.2
0.0
Xipes0191
8.9
Xpsmp2274
28.9
Xipes0157
42.1
Xicmp3027
43.7
Xipes0230
58.0
Xipes0093
62.2
Xpsmp2219
66.8
Xipes0152.2
73.5
Xpsmp2064
77.0
Xipes0214
80.1
Xipes0175
81.8
Xipes0217
84.1
Xpsmp2078
87.1
Xpsmp2229.1
96.1
Xpsmp2261
99.4
Xpsmp2233
102.4
Xpsmp2276
102.9
Xpsmp2277
104.0
Xpsmp2208
104.5
Xipes0089
113.5
Xipes0223
134.5
Si_3
Xipes0191
0.6
Bd_1
Xipes0152.2
13.0
Xipes0214
27.5
Xipes0175
63.0
Xipes0089
72.7
Xipes0223
74.0
Zm_01
Xipes0175
52.7
Xipes0152.2
74.0
Si_4
Xipes0157
34.4
Xicmp3027
43.4
Xipes0093
44.5
Os_03
Xipes0223
0.6
Xipes0089
2.1
Xipes0175
11.8
Xipes0214
13.3
Xipes0152.2
19.5
Si_9
Xipes0152.2
46.5
Xipes0214
50.8
Xipes0175
52.4
Xipes0089
63.1
Xipes0223
65.5
SBI-01
Xipes0152.2
55.0
Xipes0214
58.8
Xipes0175
60.3
Xipes0089
70.9
Xipes0223
72.9
Zm_09
Xipes0214
124.6
Xipes0089
147.5

## Slide 7
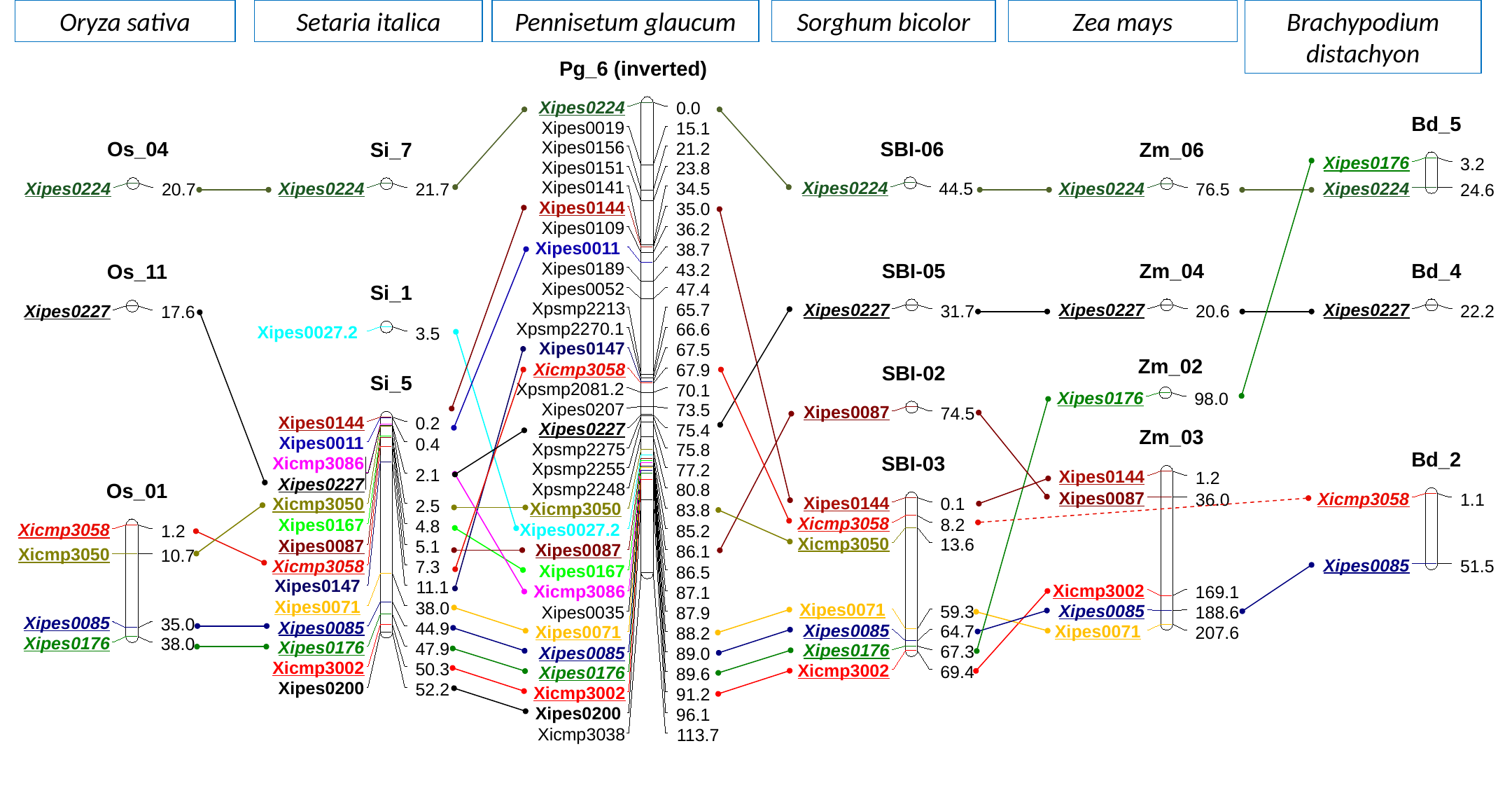

Oryza sativa
Setaria italica
Pennisetum glaucum
Sorghum bicolor
Zea mays
Brachypodium distachyon
Pg_6 (inverted)
Xipes0224
0.0
Xipes0019
15.1
Xipes0156
21.2
Xipes0151
23.8
Xipes0141
34.5
Xipes0144
35.0
Xipes0109
36.2
Xipes0011
38.7
Xipes0189
43.2
Xipes0052
47.4
Xpsmp2213
65.7
Xpsmp2270.1
66.6
Xipes0147
67.5
Xicmp3058
67.9
Xpsmp2081.2
70.1
Xipes0207
73.5
Xipes0227
75.4
Xpsmp2275
75.8
Xpsmp2255
77.2
Xpsmp2248
80.8
Xicmp3050
83.8
Xipes0027.2
85.2
Xipes0087
86.1
Xipes0167
86.5
Xicmp3086
87.1
Xipes0035
87.9
Xipes0071
88.2
Xipes0085
89.0
Xipes0176
89.6
Xicmp3002
91.2
Xipes0200
96.1
Xicmp3038
113.7
Bd_5
Xipes0176
3.2
Xipes0224
24.6
SBI-06
Xipes0224
44.5
Os_04
Xipes0224
20.7
Si_7
Xipes0224
21.7
Zm_06
Xipes0224
76.5
SBI-05
Xipes0227
31.7
Zm_04
Xipes0227
20.6
Bd_4
Xipes0227
22.2
Os_11
Xipes0227
17.6
Si_1
Xipes0027.2
3.5
Zm_02
Xipes0176
98.0
SBI-02
Xipes0087
74.5
Si_5
Xipes0144
0.2
Xipes0011
0.4
Xicmp3086
2.1
Xipes0227
Xicmp3050
2.5
Xipes0167
4.8
Xipes0087
5.1
Xicmp3058
7.3
Xipes0147
11.1
Xipes0071
38.0
Xipes0085
44.9
Xipes0176
47.9
Xicmp3002
50.3
Xipes0200
52.2
Zm_03
Xipes0144
1.2
Xipes0087
36.0
Xicmp3002
169.1
Xipes0085
188.6
Xipes0071
207.6
Bd_2
Xicmp3058
1.1
Xipes0085
51.5
SBI-03
Xipes0144
0.1
Xicmp3058
8.2
Xicmp3050
13.6
Xipes0071
59.3
Xipes0085
64.7
Xipes0176
67.3
Xicmp3002
69.4
Os_01
Xicmp3058
1.2
Xicmp3050
10.7
Xipes0085
35.0
Xipes0176
38.0

## Slide 8
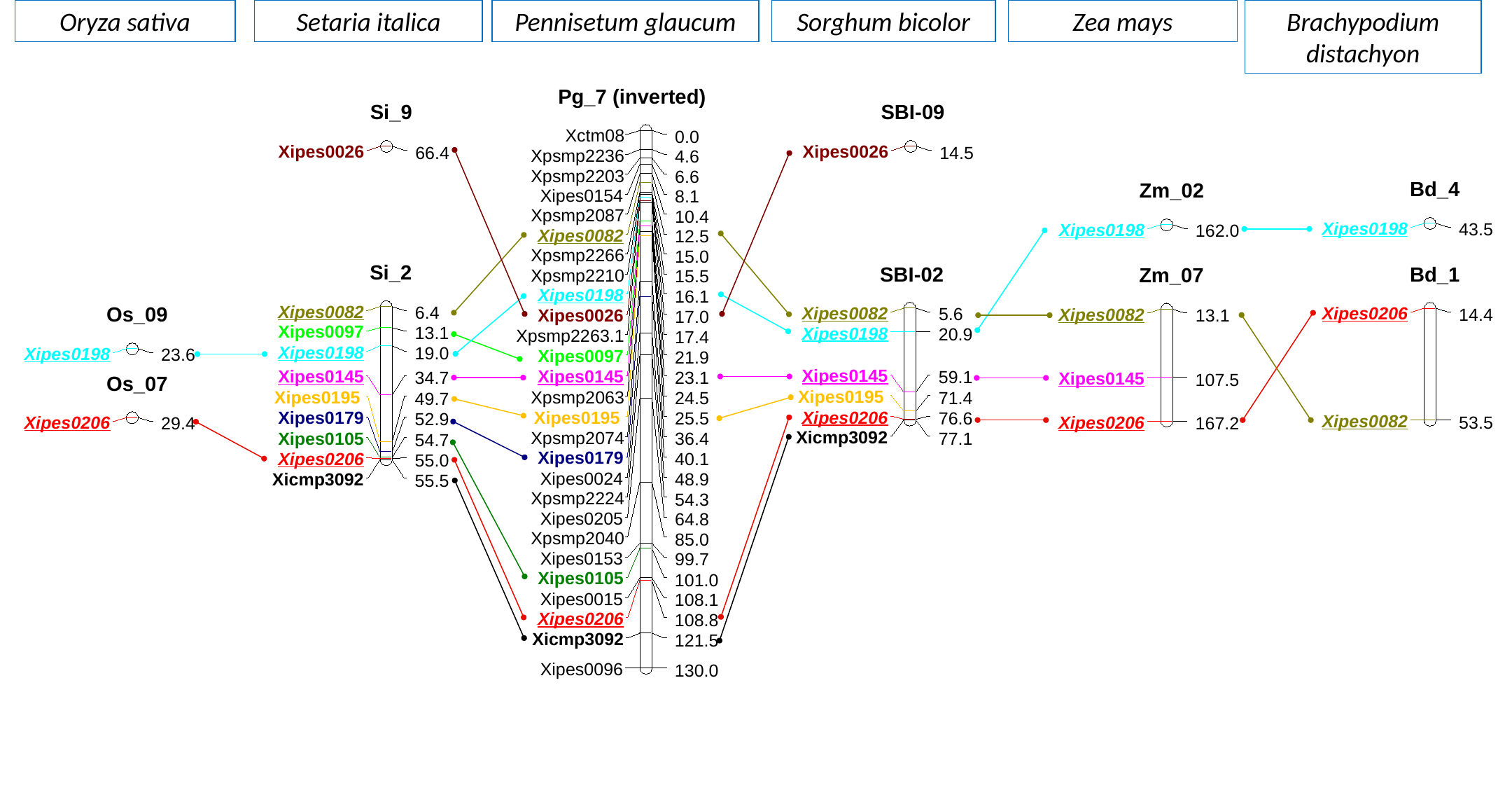

Oryza sativa
Setaria italica
Pennisetum glaucum
Sorghum bicolor
Zea mays
Brachypodium distachyon
Pg_7 (inverted)
Xctm08
0.0
Xpsmp2236
4.6
Xpsmp2203
6.6
Xipes0154
8.1
Xpsmp2087
10.4
Xipes0082
12.5
Xpsmp2266
15.0
Xpsmp2210
15.5
Xipes0198
16.1
Xipes0026
17.0
Xpsmp2263.1
17.4
Xipes0097
21.9
Xipes0145
23.1
Xpsmp2063
24.5
Xipes0195
25.5
Xpsmp2074
36.4
Xipes0179
40.1
Xipes0024
48.9
Xpsmp2224
54.3
Xipes0205
64.8
Xpsmp2040
85.0
Xipes0153
99.7
Xipes0105
101.0
Xipes0015
108.1
Xipes0206
108.8
Xicmp3092
121.5
Xipes0096
130.0
Si_9
Xipes0026
66.4
SBI-09
Xipes0026
14.5
Bd_4
Xipes0198
43.5
Zm_02
Xipes0198
162.0
Si_2
Xipes0082
6.4
Xipes0097
13.1
Xipes0198
19.0
Xipes0145
34.7
Xipes0195
49.7
Xipes0179
52.9
Xipes0105
54.7
Xipes0206
55.0
Xicmp3092
55.5
SBI-02
Xipes0082
5.6
Xipes0198
20.9
Xipes0145
59.1
Xipes0195
71.4
Xipes0206
76.6
Xicmp3092
77.1
Bd_1
Xipes0206
14.4
Xipes0082
53.5
Zm_07
Xipes0082
13.1
Xipes0145
107.5
Xipes0206
167.2
Os_09
Xipes0198
23.6
Os_07
Xipes0206
29.4
